# Role of pulmonary epithelial arginase‐II in activation of fibroblasts and lung inflammaging

**DOI:** 10.1111/acel.13790

**Published:** 2023-02-15

**Authors:** Cui Zhu, Duilio M. Potenza, Yang Yang, Guillaume Ajalbert, Kirsten D. Mertz, Stephan von Gunten, Xiu‐Fen Ming, Zhihong Yang

**Affiliations:** ^1^ Laboratory of Cardiovascular and Aging Research, Department of Endocrinology, Metabolism, and Cardiovascular System, Faculty of Science and Medicine University of Fribourg Fribourg Switzerland; ^2^ Institute for Pathology Cantonal Hospital Baselland Liestal Switzerland; ^3^ Institute of Pharmacology University of Bern Bern Switzerland

**Keywords:** aging, arginase, fibrosis, IL‐1β, inflammation, lung, TGFβ1

## Abstract

Elevated arginases including type‐I (Arg‐I) and type‐II isoenzyme (Arg‐II) are reported to play a role in aging, age‐associated organ inflammaging, and fibrosis. A role of arginase in pulmonary aging and underlying mechanisms are not explored. Our present study shows increased Arg‐II levels in aging lung of female mice, which is detected in bronchial ciliated epithelium, club cells, alveolar type 2 (AT2) pneumocytes, and fibroblasts (but not vascular endothelial and smooth muscle cells). Similar cellular localization of Arg‐II is also observed in human lung biopsies. The age‐associated increase in lung fibrosis and inflammatory cytokines, including IL‐1β and TGF‐β1 that are highly expressed in bronchial epithelium, AT2 cells, and fibroblasts, are ameliorated in *arg‐ii* deficient (*arg‐ii* ^*−/−*^) mice. The effects of *arg‐ii* ^*−*/−^ on lung inflammaging are weaker in male as compared to female animals. Conditioned medium (CM) from human Arg‐II‐positive bronchial and alveolar epithelial cells, but not that from *arg‐ii* ^*−/−*^ cells, activates fibroblasts to produce various cytokines including TGF‐β1 and collagen, which is abolished by IL‐1β receptor antagonist or TGF‐β type I receptor blocker. Conversely, TGF‐β1 or IL‐1β also increases Arg‐II expression. In the mouse models, we confirmed the age‐associated increase in IL‐1β and TGF‐β1 in epithelial cells and activation of fibroblasts, which is inhibited in *arg‐ii* ^*−*/−^ mice. Taken together, our study demonstrates a critical role of epithelial Arg‐II in activation of pulmonary fibroblasts via paracrine release of IL‐1β and TGF‐β1, contributing to pulmonary inflammaging and fibrosis. The results provide a novel mechanistic insight in the role of Arg‐II in pulmonary aging.

## Graphical abstract

Our study demonstrates a critical role of the mitochondrial arginase type II (Arg‐II) in pulmonary inflammaging and fibrosis through cell autonomous and paracrine mechanisms. In lung epithelial cells, Arg‐II promotes IL‐1beta and TGF‐beta1 expression and release, which activates fibroblasts to express Arg‐II and inflammatory cytokines as well as to accumulate matrix. The results provide a novel mechanistic insight in the role of Arg‐II in pulmonary aging and implicate a therapeutic potential of targeting Arg‐II in pulmonary inflammaging and fibrosis.

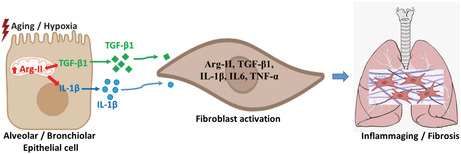

**Abbreviations**
Arg‐I: arginase isoform I
Arg‐II: arginase isoform II
arg‐ii^‐/‐^
: Arg‐II knock out
AT2: alveolar type 2 (pneumocytes)
CC‐10: Clara Cells and Clara Cell 10 kD Protein (marker of Club cells / Clara cells)
CM: conditioned medium
COPD: chronic obstructive pulmonary disease
DAPI: 4′,6‐diamidino‐2‐phenylindole
FBS: fetal bovine serum
FOXJ1: Forkhead Box J1 (marker of bronchial ciliated epithelial cells)
GAPDH: glyceraldehyde 3‐phosphate dehydrogenase
IH: intermittent hypoxia
IL‐1β: interleukin‐1β
IL‐1Ra: interleukin‐1 receptor antagonist
ΙL−6: interleukin‐6
iNOS: inducible nitric oxide synthase
MCP1: monocyte chemoattractant protein‐1
ΝΟ: nitric oxide
ΝΟS: nitric oxide synthase
PMSF: phenylmethylsulfonyl fluoride
rps12: ribosomal protein S12
sdha: succinate dehydrogenase complex flavoprotein subunit A
SMA: smooth muscle actin
SP‐C: surfactant protein‐C (marker of alveolar type 2 pneumocytes)
TGF‐β1: Τransforming Growth Factor‐β1
TNF‐α: tumor necrosis factor‐α
wt: wild type

## 1 INTRODUCTION

Physiological lung aging contributes to functional and structural alterations of the organ and increases its susceptibility to a wide range of pulmonary dieases, whereby chronic inflammation and fibrosis are involved (Gulati & Thannickal, 2019; Schneider et al., 2021). Moreover, advancing aging is associated with increase in incidence of sleep apnea that causes chronic hypoxia or chronic intermittent hypoxia which has been shown to accelerate pulmonary diseases (Gille et al., 2018; Lee et al., 2019). To further explore molecular mechanisms of physiological lung aging could provide novel insight in understanding pulmonary aging process and lead to identification of novel therapeutic targets for age‐associated lung diseases.

The lung is composed of over 40 discrete cell types, including various respiratory epithelial cells, lung stem and/or progenitor cells, pulmonary immune cells, vascular cells, and fibroblasts, etc. (Schiller et al., 2019). Lung aging alone without diseases is characterized by alterations of bulk gene expression patterns, decreased respiratory epithelial cells with concurrent activation of inflammatory cells, impaired regenerative capacity, and remodeling of lung tissue extracellular matrix and increase in tissue stiffness (Angelidis et al., 2019; Schneider et al., 2021). Although many mechanisms of lung aging are discovered and comprehensively reviewed by Schneider et al. (2021), the underlying cellular and molecular mechanisms are still poorly understood. It is essential to investigate the mechanisms of molecular and cellular functional changes and crosstalk between the discrete cell types in pulmonary aging, which contributes to susceptibility to pulmonary diseases.

In the past years, there are substantial studies demonstrating a crucial role of the enzyme arginase in aging phenotype of various organs, including heart, blood vessels, skin, pancreas, and kidneys (Berkowitz et al., 2003; Huang et al., 2021; Xiong et al., 2014; Xiong, Yepuri, Montani, et al., 2017; Xiong, Yepuri, Necetin, et al., 2017; Yepuri et al., 2012). Arginase, the ureohydrolase that metabolizes L‐arginine to urea and L‐ornithine, has two isoforms, that is, the cytoplasmic arginase‐I (Arg‐I) and the mitochondrial arginase‐II (Arg‐II), which are encoded by different genes (Jenkinson et al., 1996). In addition to the well‐known vital role of Arg‐I in ammonia detoxification through urea cycle that takes place mainly in the liver, the functions of Arg‐I and Arg‐II in extrahepatic tissues are mainly attributed to the decrease in L‐arginine bioavailability and the increase in L‐ornithine with downstream production of polyamines and L‐proline (Yang & Ming, 2006). The reduction of L‐arginine bioavailability for nitric oxide synthase (NOS) leads to decreased NO production in endothelial cells, while the downstream metabolite L‐proline and polyamines are involved in collagen synthesis and cell proliferation, respectively (Yang & Ming, 2006). Moreover, enzymatic activity‐independent functions of Arg‐II have been reported (Xiong et al., 2013, 2014). Studies from past years provide evidence suggesting that Arg‐II in particular, plays a causal role in organ/organismal aging at least partly through stimulation of chronic inflammatory responses and tissue fibrosis (Huang et al., 2021) (Endo et al., 2003). It is, however, not clear, whether arginase and which isoform of the enzyme is involved in pulmonary aging that is associated with inflammation (inflammaging) and fibrosis, although both isoforms of arginase Arg‐I and Arg‐II have been reported to play a role in the pathogenesis of pulmonary diseases, including asthma, cystic fibrosis, and chronic obstructive pulmonary disease (COPD) (Jaecklin et al., 2014; Maarsingh et al., 2008; Pera et al., 2014). Moreover, a recent study demonstrates that arginase activity and Arg‐II protein level are increased in bleomycin‐induced pulmonary fibrosis of a mouse model and inhibition of arginase activity reveals significant therapeutic effect on pulmonary fibrosis (Gao et al., 2019). Importantly, cellular localization of arginases and how the arginase‐expressing cells contribute to aging‐associated pulmonary inflammation and fibrosis are not known.

One of the key cytokines involved in pulmonary inflammation and fibrosis is transforming growth factor‐β1 (TGF‐β1) (Munger et al., 1999; Ong et al., 2021), whose expression and activity have been shown to be increased in pulmonary fibrosis in advanced aging (Ramirez et al., 2007; Sueblinvong et al., 2012). Interestingly, in a mouse lung allograft fibrosis model, TGF‐β1 increases both Arg‐I and Arg‐II expression, suggesting an interaction between TGF‐β1 and arginase in lung diseases (Liu et al., 2005). Our previous studies have demonstrated that age‐associated augmentation of Arg‐II (not Arg‐I) levels in various organs in mouse model plays an important role in organ/tissue dysfunction and aging phenotypes (Huang et al., 2021; Xiong, Yepuri, Necetin, et al., 2017; Yepuri et al., 2012). A role of Arg‐II and its interaction with TGF‐β1 in aging‐associated pulmonary fibrosis have not been explored, yet.

Hence the present study aims to investigate a role of arginase in pulmonary inflammaging and fibrosis. Cellular localization of arginases, and mechanisms of elevated arginase in pulmonary inflammaging and fibrosis via crosstalk between epithelial cells and fibroblasts are explored.

## 2 RESULTS

### 2.1 Increased Arg‐II expression levels in aging lung of female mice

**FIGURE 1:**
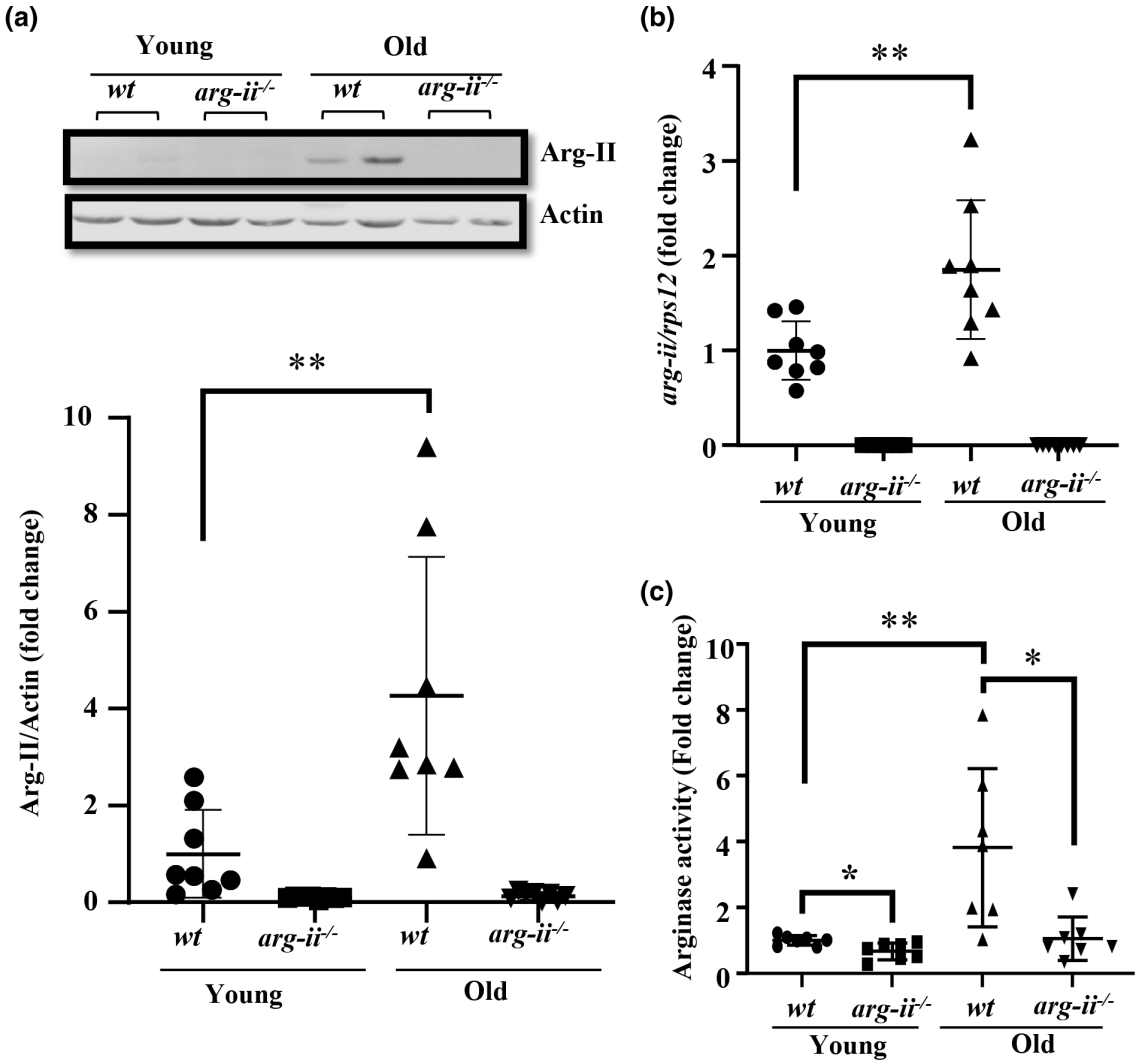
Age‐associated increase in Arg‐II levels in female mouse lung. (a) Immunoblotting analysis of Arg‐II in the lung of female *wt* and *arg‐ii* ^*−/−*^ mice. β‐Actin served as loading control. Lower panel represents plot graphs showing the quantification of the signals on immunoblots (*n* = 8 mice in each group). (b) *arg‐ii* mRNA levels of female mouse lung tissues analyzed by qRT‐PCR. *rps12* served as the reference (*n* = 8 animals in each group). (c) Arg‐II activity analysis in the lung of female *wt* and *arg‐ii* ^*−/−*^ mice. (*n* = 7 mice in each group). The values shown are mean ± SD. Data are presented as the fold change to the young‐*wt* group. *p ≤ 0.05, **p ≤ 0.01 between the indicated groups. *wt*, wild‐type mice; *arg‐ii* ^*−/−*^, *arg‐ii* gene knockout mice.

To study the role of arginase in pulmonary aging, expression of Arg‐I and Arg‐II at both mRNA and protein levels in lungs from young (3–4 months in age) and old (22–24 months in age) mice is first examined. Immunoblotting analysis of lungs from female mice reveals an age‐associated elevation of Arg‐II protein levels (Figure 1a) in the *wt* mice, which is accompanied by an increase in *arg‐ii* mRNA levels as analyzed by qRT‐PCR (Figure 1b). Moreover, an age‐associated increase in arginase enzymatic activity in the lung is observed in *wt* mice but not in *arg‐ii* ^−/−^ animals (Figure 1c). These results suggest that the age‐associated increase in arginase enzymatic activity in lung is due to elevated Arg‐II levels. In accordance, no Arg‐I protein could be detected in lung tissues by immunoblotting in young and old mice of both genotypes (Figure S1a). Although *arg‐i* mRNA is detected in the lung with low Ct value (data not shown), there are, however, no significant differences among the young and old mice of both *wt* and *arg‐ii* ^−/−^ (Figure S1b). The specificity of the anti‐Arg‐I antibody is validated with liver tissue used as positive control as shown in Figure S1a. Furthermore, the very low expression levels of Arg‐I in mouse lung tissue are further confirmed by comparing with mouse liver samples (Figure S1c). These data indicate that Arg‐I is expressed at very low level in lung, which could be detected only by sensitive qRT‐PCR, but not by immunoblotting. In contrast to females, male animals do not show age‐associated increase in Arg‐II protein (Figure S2b) and mRNA levels (Figure S2b). Also, no age‐associated increase in arginase enzymatic activity is found in the lung of male mice (Figure S2c), whereas *arg‐ii* ^−/−^ mice show decreased arginase enzymatic activity in the lung of both young and old males as expected (Figure S2c). No difference in *arg‐i* gene expression in the lungs between males and females is observed (Figure S2d). Therefore, further studies are focused on Arg‐II and female mice.

### 2.2 *Arg‐ii* gene ablation suppresses age‐associated lung fibrosis and inflammation

**FIGURE 2:**
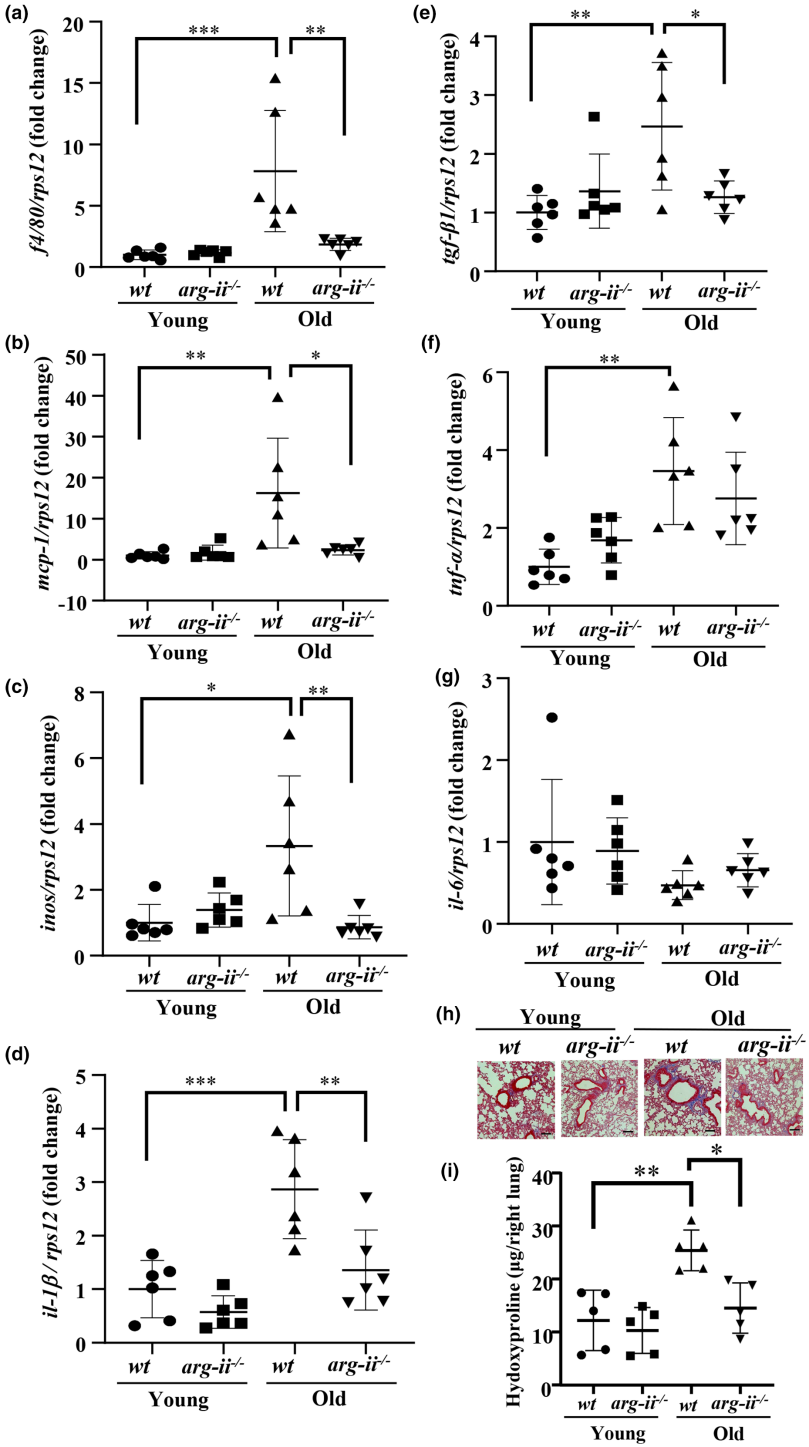
Age‐associated elevation in inflammatory cytokines in the lung was prevented in female *arg‐ii* ^*−/−*^ mice. mRNA expression levels of (a) *f4/80*, (b) *mcp‐1*, (c) *inos*, (d) *il‐1β*, (e) *tgf‐β1*, (f) *tnf‐α*, and (g) *il‐6* in young and old *wt* and *arg‐ii* ^*−/−*^ female mouse lung were analyzed by qRT‐PCR. rps12 served as the reference (*n* = 6 mice in each group). The values shown are mean ± SD. Data are expressed as the fold change to the young *wt* group. (h) Representative histological images of lung interstitial fibrosis in young and old *wt* and *arg‐ii* ^*−/−*^ female mice. Fibrosis is shown by the blue‐colored Trichrome Masson's staining. Scale bar = 100 μm (*n* = 4 mice in each group). (i) Hydroxyproline content of mouse right lung from young and old *wt* and *arg‐ii* ^*−/−*^ female mice (*n* = 5 mice in each group). *p ≤ 0.05, **p ≤ 0.01, and ***p ≤ 0.005 between the indicated groups. *wt*, wild‐type mice; *arg‐ii* ^*−/−*^, *arg‐ii* gene knockout mice.

Masson's trichrome staining reveals increased peri‐bronchiolar fibrosis in the *wt* old mice with occasional fibrotic foci in pulmonary parenchyma, which is mitigated in the age‐matched *arg‐ii* ^*−/−*^ animals (Figure 2h and Figure S3). The increased lung fibrosis in aging is further confirmed by quantitative measurement of elevated hydroxyproline levels in *wt* old mice as compared to the young animals (Figure 2i). This age‐associated increase in hydroxyproline levels is inhibited in the age‐matched *arg‐ii* ^*−/−*^ mice (Figure 2i), demonstrating that *arg‐ii* gene ablation prevents age‐associated pulmonary fibrosis. Moreover, gene expression of a series of pro‐inflammatory cytokines such as the pan‐macrophage marker *f4/80*, *mcp1*, *inos*, *il‐1β*, *tgf‐β1*, *tnf‐α*, but not *il‐6* are significantly increased in old *wt* lung tissues (Figure 2). The age‐associated increases in these inflammatory cytokines (except *tnf‐α*, Figure 2f and *il‐6*, 2g) are significantly decreased or prevented in *arg‐ii* ^*−/−*^ mice (Figure 2a–e). The cell senescence markers *p16* ^*ink4*^ and *p21* ^*Cip1*^ are significantly increased in aging mice and tended to be lower in age‐matched *arg‐ii* ^*−/−*^ animals (Figure S4). In contrast to female mice, the effect of *arg‐ii* ^*−/−*^ on age‐associated pulmonary inflammation is weaker in male animals. Only the age‐associated increases in *f4/80* levels (Figure S5) and the senescent marker *p16* ^*ink4*^ (Figure S6) are significantly inhibited in the *arg‐ii* ^*−/−*^ mice. Similar to female mice, age‐associated increase in pulmonary fibrosis as assessed by hydroxyproline content in the lung of male mice is significantly decreased in the age‐matched *arg‐ii* ^*−/−*^ mice (Figure S5h).

### 2.3 Cellular localization of Arg‐II in aging lung

**FIGURE 3:**
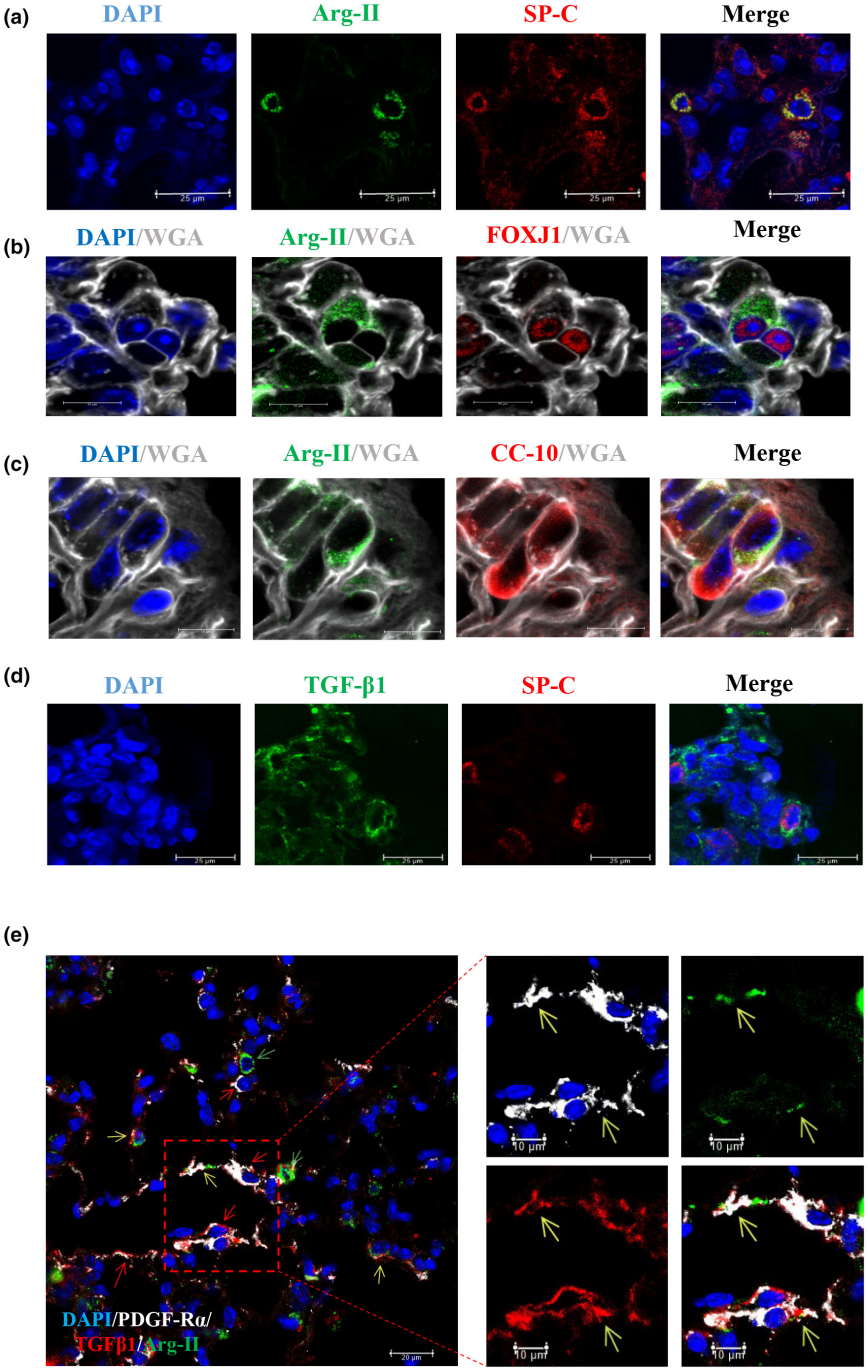
Cellular localization of Arg‐II and TGF‐β1 expression in aging lung of female mice. Confocal microscopy illustration of immunofluorescence double staining of (a) Arg‐II (green) and SP‐C (red, alveolar type II pneumocyte marker). Scale bar = 25 μm; (b) Arg‐II (green) and FOXJ1 (red, bronchiolar ciliated epithelium marker). WGA (grey) stains cell membrane and DAPI (blue) stains cell nucleoli in aging female mouse lung tissues. Scale bar = 10 μm; (c) Arg‐II (green) and CC‐10 (red, bronchiolar club cell marker) in aging female mouse lung tissues. WGA (grey) stains cell membrane and DAPI (blue) stains cell nucleoli. Scale bar = 10 μm. This experiment was repeated with five animals. Confocal microscopy illustration of immunofluorescence double staining of (d) TGF‐β1 (green) and SP‐C (red, alveolar type II pneumocyte marker). Scale bar = 25 μm; (e) TGF‐β1 (red), PDGF‐Rα (white) and Arg‐II (green) in alveolar region. Yellow arrows indicate Arg‐II^+^/TGF‐β1^+^/PDGF‐Rα^+^ cells; red arrows indicate TGF‐β1^+^/PDGF‐Rα^+^ but Arg‐II^−^ cells; green arrows indicate cells positive only to Arg‐II staining. DAPI (blue) stains cell nucleoli. Scale bar = 20 μm. This experiment was repeated with five animals.

We then further examined what types of cells in the lung express Arg‐II. As Arg‐II level is not generally upregulated in aging but in female mice alone, lung tissues from old female mice were used. Co‐immunostaining of lung sections demonstrates that Arg‐II is present in cells expressing SP‐C (marker of alveolar type 2 pneumocytes or AT2 cells, Figure 3a). Co‐immunostaining also shows that Arg‐II is expressed in FOXJ1‐positive cells, that is, bronchial ciliated epithelial cells (Figure 3b) and in CC‐10‐positive cells, that is, Club cells (also known as Clara cells, Figure 3c). The cell border is marked by WGA which stains for cell membrane. With a human normal lung biopsy tissue obtained from a 68‐year‐old man, Arg‐II is found in AT2 cells as demonstrated by co‐staining with SP‐C (Figure S7a). Single cell RNA‐sequencing from Human Protein Atlas Database shows that Arg‐II is expressed in various cell types in human lung including AT2, Club cells, ciliated cells, fibroblasts, immune cells, but not in endothelial cells and AT1 cells (Figure S7b) (https://www.proteinatlas.org/ENSG00000081181‐ARG2/single+cell+type/lung), which is in line with our findings in mouse model.

### 2.4 Cellular localization of TGF‐β1 in aging lung

As TGF‐β1 plays an important role in tissue fibrosis and is increased in aging lung tissues and decreased in *arg‐ii* ^*−/−*^ mice as shown in Figure 2, further experiments investigated cellular localization of TGF‐β1 in aging lung and its relationship with Arg‐II. Co‐immunostaining analysis reveals that TGF‐β1 is expressed in alveolar AT2 cells expressing SP‐C (Figure 3d) and in fibroblasts of bronchioles as demonstrated by immunofluorescence triple staining of PDGF‐Rα, TGF‐β1, and Arg‐II (Figure 3e). It is to note that Arg‐II and TGF‐β1 are not detectable in vascular endothelial cells as shown by co‐staining experiments with CD31 (Figure S8a,c respectively) and very few vascular smooth muscle cells express TGF‐β1 as shown by co‐staining with α‐SMA (Figure S8d).

### 2.5 *Arg‐ii* gene ablation suppresses TGF‐β1 levels in lung

**FIGURE 4:**
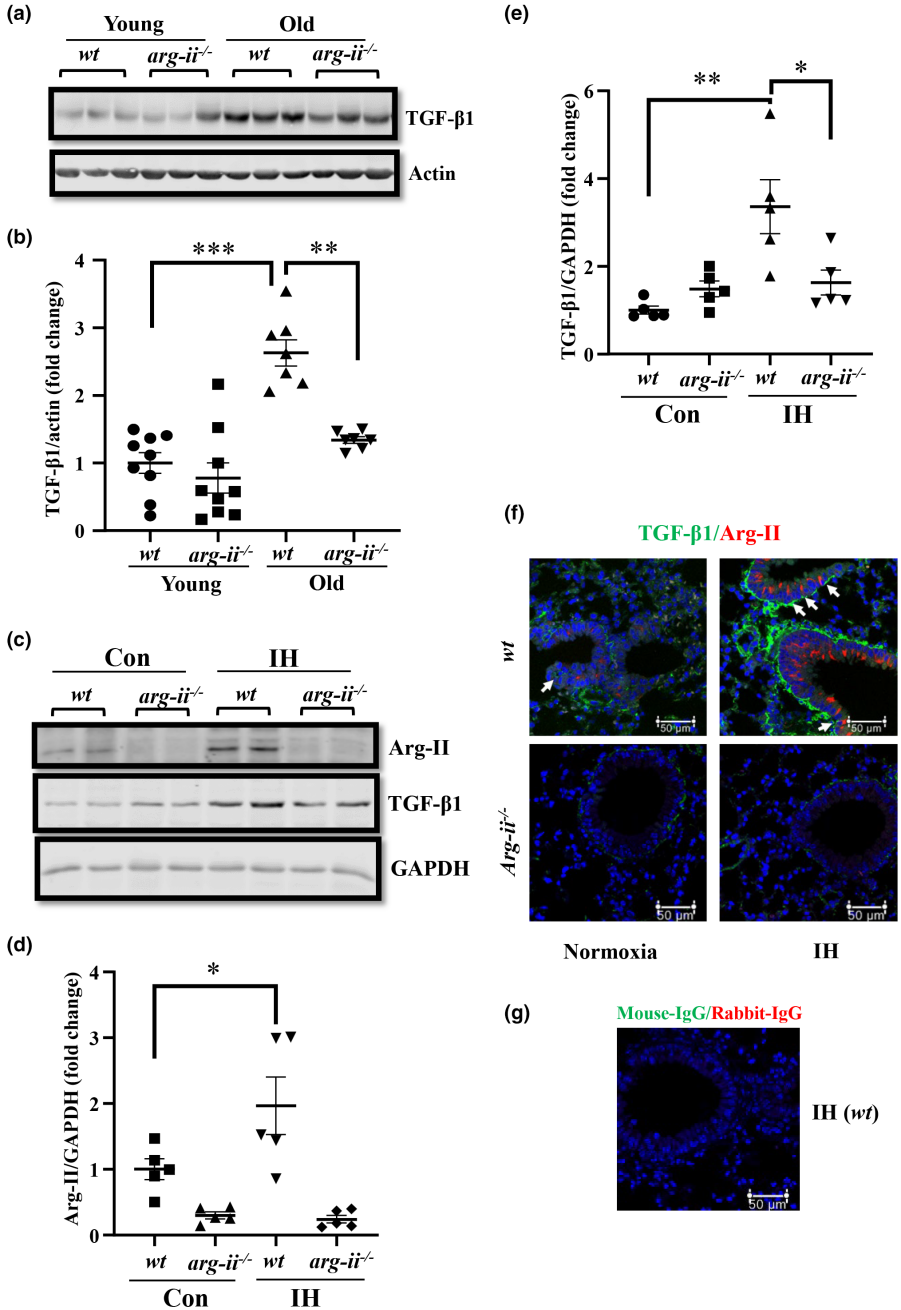
Arg‐II upregulates TGF‐β1 in vitro and in vivo models. *Arg‐ii* deficiency decreases age‐associated increase in TGF‐β1 in the lung of female mice. (a) Immunoblotting analysis of TGF‐β1 in the lung of female young (3–4 months) and old (22–24 months) mice, β‐Actin served as protein loading controls; (b) The plot graphs show the quantification of the signals on immunoblots (*n* = 7–9 mice in each group); (c) Immunoblotting analysis of Arg‐II and TGF‐β1 in female *wt* and *arg‐ii* ^*−/−*^ mice under intermittent hypoxic (IH) conditions for 21 days and normoxic conditions as controls (Con). GAPDH is used as protein loading control, as β‐Actin is unstable under hypoxic conditions; (d,e) Plot graphs show the quantification of the signals of Arg‐II and TGF‐β1 on the immunoblots (*n* = 5 mice in each group); (f) A representative confocal microscopy of immunofluorescence co‐staining of TGF‐β1 and Arg‐II in the lung of a mouse exposed to normoxia and intermittent hypoxia (IH) for 21 days; (*n* = 5 mice in each group). (g) Negative control (Anti‐mouse‐IgG) of immunofluorescence staining in the lung of a *wt* mouse exposed to IH. Data are presented as fold change to the normoxic young *wt* group. *p ≤ 0.05, **p ≤ 0.01, and ***p ≤ 0.005 between the indicated groups. *wt*, wild‐type mice; *arg‐ii* ^*−/−*^, *arg‐ii* gene knockout mice. IH, intermittent hypoxia. Scale bar = 50 μm.

In line with the mRNA levels shown in Figure 2, TGF‐β1 protein levels are also increased in the lung of old *wt* mice, which is decreased in age‐matched *arg‐ii* ^*−/−*^ animals (Figure 4a,b), implicating that TGF‐β1 expression is regulated by Arg‐II in aging. This local regulation of TGF‐β1 by Arg‐II in aging lung is not accompanied by corresponding changes in circulating serum levels of the cytokine as measured by ELISA (Figure S9). Similarly, intermittent hypoxia for 21‐days is able to increased Arg‐II and TGF‐β1 levels in the lung of young animals, which is inhibited in *arg‐ii* ^*−/−*^ mice (Figure 4c–e), and also under hypoxic conditions when Arg‐II is upregulated. Immunofluorescence confocal microscopy demonstrates that intermittent hypoxia (IH) increases Arg‐II levels in bronchiolar epithelial cells as well as in alveolar AT2 peumocytes in *wt* mice (Figure 4f). The levels of TGF‐β1 were found to be increased under hypoxic conditions in AT2 cells and largely accumulated at the basal membranes surrounding bronchiolar epithelial cells expressing Arg‐II (Figure 4f). The increase in TGF‐β1 under this condition is inhibited in *arg‐ii* ^*−/−*^ mice (Figure 4f). These results implicate that Arg‐II upregulates TGF‐β1 levels at least in the lung AT2 cells and likely in bronchiolar epithelial cells. The accumulation of TGF‐β1 at the basal membrane side surrounding bronchioles suggests that TGF‐β1 might be produced from epithelial cells and secreted to the abluminal side of the bronchioles. With pulmonary epithelial cells in culture, we demonstrate that TGF‐β1 is indeed released from the cells and activates fibroblasts (please see below).

### 2.6 Crosstalk between epithelial cells and fibroblasts: Role of TGF‐β1 and IL‐1β

**FIGURE 5:**
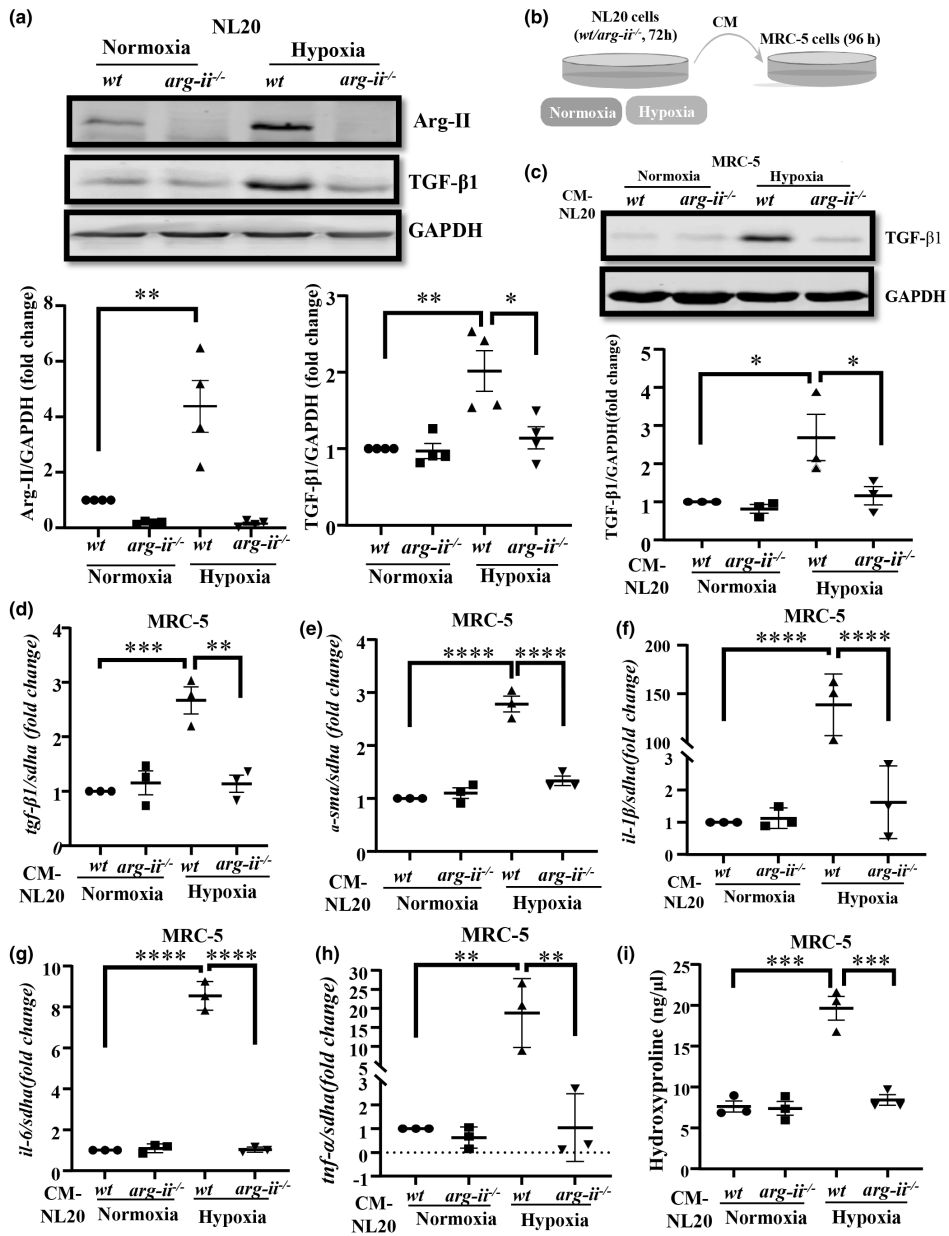
*Arg‐II*‐positive human bronchial epithelial cells (NL‐20) activates fibroblasts. (a) Immunoblotting analysis of Arg‐II and TGF‐β1 in NL‐20 cells. GAPDH as protein loading control, as β‐Actin is unstable under hypoxic conditions. The plot graphs below show the quantification of the signals on immunoblots (*n* = 4 independent experiments); (b) Schematic illustration of the experimental setup to study crosstalk between epithelial cells and fibroblasts. Human bronchial epithelial cells (NL‐20‐CRISPR‐*wt* and ‐CRISPR‐*arg‐ii* ^*−/−*^) were exposed to either normoxia or hypoxia (1% O_2_) conditions for 72 h. Conditioned medium (CM) was then collected from NL20 cells and transferred to fibroblasts (MRC‐5) for 96 h. Cell lysates and RNA of MRC‐5 were then prepared and subjected to immunoblotting and qRT‐PCR analysis, respectively. (c) Immunoblotting analysis of TGF‐β1 in MRC‐5 cells, GAPDH as protein loading control. The plot graphs show the quantification of the signals on immunoblots (*n* = 3 independent experiments). (d–h) qRT‐PCR analysis of mRNA expression levels of (d) *tgf‐β1*, (e) *α‐sma*, (f) *il‐1β*, (g) il‐6, and (h) *tnf‐a* in MRC‐5 cells. *sdha* served as the reference (*n* = 3 independent experiments). (i) Collagen production measured by hydroxyproline in MRC‐5 cells (*n* = 3 independent experiments). The values shown are mean ± SD. Data are expressed as the fold change to the normoxic *wt* group (panels a, c–h). *p ≤ 0.05, **p ≤ 0.01, ***p ≤ 0.005, and ****p ≤ 0.001 between the indicated groups. *arg‐ii* ^*−/−*^, arg‐ii knockout NL‐20 cell by CRISPR; *wt*, wild‐type NL‐20 cells. NL20‐CM, NL‐20 cell conditioned medium.

As increased Arg‐II levels in aging lung and/or under hypoxic conditions are found in the epithelial cells, including bronchiolar epithelial cells and AT2 pneumocytes, and that TGF‐β1 is detected in fibroblasts expressing Arg‐II (Figure 3e) and substantially accumulated in the abluminal side in proximity to Arg‐II‐positive epithelial cells (Please note that vascular endothelial cells in aging do not express much Arg‐II or TGF‐β1, only few vascular SMC express TGF‐β1, Figure S8d), we hypothesize that the Arg‐II upregulation in the bronchiolar epithelial cells and AT2 cells may activate pulmonary fibroblasts through a paracrine mechanism. For this purpose, *wt* and *arg‐ii* ^*−/−*^ human epithelial cells (NL20 cells) are exposed to hypoxia (1% O_2_ for 72 h) to increase Arg‐II. As shown in Figure 5a, hypoxia significantly up‐regulates Arg‐II and also TGF‐β1 level in the *wt* NL20 cells and deficiency in *arg‐ii* gene (*arg‐ii* ^*−/−*^) in the cells decreases TGF‐β1 level under hypoxic conditions. Importantly, human lung fibroblasts (MRC5) stimulated with conditioned medium from hypoxic NL‐20 epithelial cells for 96 h (Figure 5b), exhibit elevated TGF‐β1 protein (Figure 5c) and mRNA levels (Figure 5d) accompanied with increased mRNA levels of *α‐sma*, *il‐1β*, *il‐6*, and *tnf‐α* (Figure 5e–h), which is abolished when *arg‐ii* gene in epithelial cells is ablated (Figure 5c–h). Hydroxyproline levels in the fibroblasts are increased by conditioned medium from hypoxic *wt* but not *arg‐ii* ^*−/−*^ NL20 cells (Figure 5i).

Similar results are also obtained with A549 cells, a widely used human alveolar AT2 pneumocyte model (Figure S10). Under hypoxic conditions (1% O_2_ for 72 h), Arg‐II and TGF‐β1 protein levels are increased in *wt* cells (Figure S10a). Conditioned medium from hypoxic *wt* A549 cells, but not *arg‐ii* ^*−/−*^ cells, increases TGF‐β1 levels in the human lung fibroblasts (MRC5) (Figure S10b,c). Elevated gene expression of *tgf‐β1*, *α‐sma*, *il‐1β*, *il‐6*, and *tnf‐α* by wt‐A549 hypoixic conditioned medium is abolished when *arg‐ii* gene in the epithelial cells is ablated (Figure S10d–h). Hydroxyproline levels in the fibroblasts are increased by conditioned medium from hypoxic *wt* but not *arg‐ii* ^*−/−*^ A549 cells (Figure S10i). These results demonstrate that Arg‐II‐positive epithelial cells activate pulmonary fibroblasts through a paracrine mechanism(s). Interestingly, TGF‐β1 is also able to increase Arg‐II protein levels in epithelial cells (NL‐20, A549) and also in fibroblasts (MRC5), which is prevented by TGF‐β receptor‐I blocker (SB431542, 10 nmol/L) (Figure S11).

**FIGURE 6:**
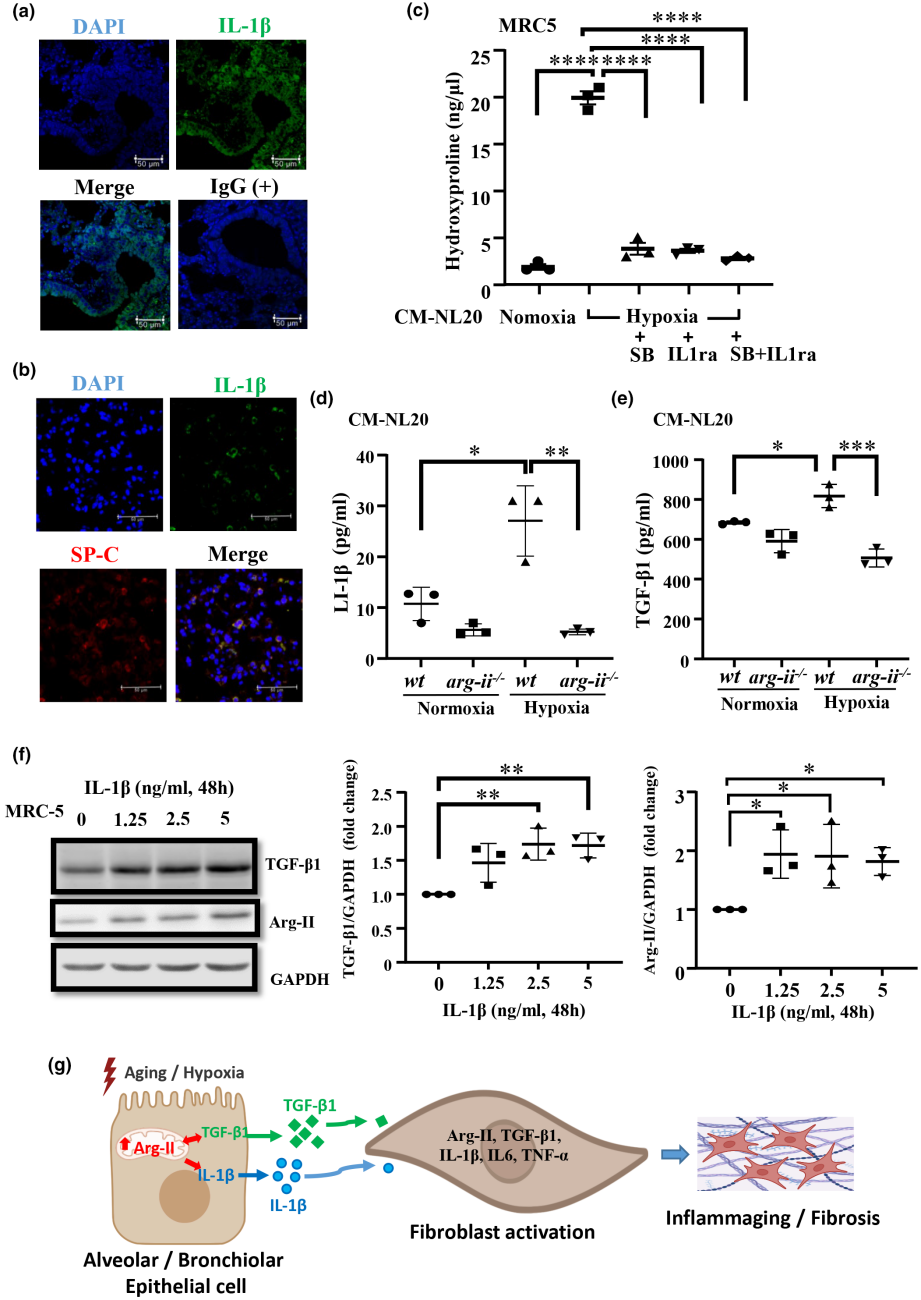
IL‐1β and TGF‐β1 as mediators in crosstalk between epithelial cells and fibroblasts. (a) Confocal microscopy of immunofluorescence staining of IL‐1β (green) in lung of old female mice; rabbit‐IgG as a negative control, DAPI stains cell nucleoli, Scale bar = 50 μm; This experiment was repeated with four animals. (b) Immunofluorescence double staining of IL‐1β (green) and SP‐C (red, alveolar type II epithelium marker) in lung of old female mice; DAPI stains cell nucleoli. Scale bar = 50 μm; This experiment was repeated with five animals. (c) Human lung fibroblasts (MRC‐5) were stimulated with conditioned medium from normoxic or hypoxic NL20 epithelial cells (CM‐NL20) as described in Figure 5b in the absence or presence of the TGF‐β1 type I receptor blocker SB431542 or IL‐1 receptor antagonist IL‐1Ra or both. Hydroxyproline levels were measured in MRC‐5 fibroblasts (*n* = 3 independent experiments); (d,e) IL‐1β and TGF‐β1 levels in the conditioned medium from normoxic or hypoxic *wt* and *arg‐ii* ^*−/−*^ NL20 epithelial cells were measured by ELISA (*n* = 3 independent experiments). (f) Immunoblotting analysis of TGF‐β1 levels in MRC5 fibroblasts. GAPDH served as protein loading controls (*n* = 3 independent experiments). Data are expressed as fold change to the control group. *p ≤ 0.05 **p ≤ 0.01, ***p ≤ 0.005, ****p ≤ 0.001 between the indicated groups. CM‐NL20, conditioned medium from NL‐20 cells. SB, SB431542, the TGF‐β1 receptor I inhibitor (10 μmol/L); IL‐1ra, interleukin 1 receptor antagonist (100 ng/mL). (g) Schematic illustration of role and mechanisms of Arg‐II in crosstalk between pulmonary epithelial cells and fibroblasts, contributing to lung fibrosis in aging.

As shown in Figure 2, one of the pro‐inflammatory cytokines increased in aging lung is IL‐1β. Therefore, we examined the cellular origin of IL‐1β in the lung. Co‐immunostaining reveals that IL‐1β is detected in bronchiolar epithelial cells (Figure 6a) and also in AT2 pneumocytes as demonstrated by co‐localization with SP‐C (Figure 6b). To examine whether IL‐1β and/or TGF‐β1 from bronchiolar epithelial cells and AT2 cells are the paracrine mediator(s) for activation of fibroblasts, experiments with conditioned medium from NL‐20 and A549 cells were performed in the absence or presence of the TGF‐β1 Type I receptor blocker SB431542 or IL‐1 receptor antagonist IL‐1Ra or both. The results demonstrate that conditioned medium from hypoxic NL20 cells increases collagen production in the MRC5 fibroblasts as measured by increase in hydroxyproline levels (Figure 6c), which is abolished either by the recombinant IL1ra, an IL‐1 receptor antagonist or by the TGF‐β1 type I receptor blocker SB431542 or the combination of both inhibitors (Figure 6c). Furthermore, ELISA experiments confirm that both IL‐1β and TGF‐β1 are significantly increased in the conditioned media from hypoxic NL20 epithelial cells as compared to the conditioned media from normoxic cells (Figure 6d,e). Similar results are also obtained with conditioned medium of A549 cells (Figure S12). Furthermore, stimulation of human fibroblasts with IL‐1β increases TGF‐β1 levels (Figure 6f), which confirms the role of epithelial cell‐derived IL‐1β in activation of fibroblasts. Interestingly, treatment of the cells with IL‐1β also increases Arg‐II levels (Figure 6f), demonstrating a positive circuit among Arg‐II, TGF‐β1, and IL‐1β in activation of the cells.

## 3 DISCUSSION

Lung aging is characterized by complex multiscale changes in structure, cellular, and molecular composition. Among them, chronic inflammation in aging lung or pulmonary inflammaging and decreased pulmonary compliance due to tissue fibrosis are the remarkable features of lung aging (Schneider et al., 2021). In the present study, we provide evidence for a crucial role of Arg‐II in pulmonary aging in female mice. This conclusion is supported by the results showing an age‐associated increase in Arg‐II expression at both mRNA and protein levels in the lung of females, which is accompanied by increased pulmonary fibrosis surrounding bronchioles and increased hydroxyproline content, and elevated pro‐inflammatory cytokines and macrophages. Ablation of Arg‐II in the mice (*arg‐ii* ^*−/−*^) decreases the inflammaging markers including pro‐fibrotic TGF‐β1 and the hydroxyproline content, demonstrating a role of Arg‐II in lung aging. The role of arginase in pulmonary fibrosis has been reported in various lung disease models, including cystic fibrosis, COPD, and bleomycin‐induced pulmonary fibrosis (Gao et al., 2019; Grasemann et al., 2005; Jaecklin et al., 2014; Maarsingh et al., 2008). The contribution of Arg‐I or Arg‐II to pulmonary diseases is, however, obscure.

Our current study is in line with previous findings showing that Arg‐II expression/activity levels are elevated in numerous organs of aged mice, and ablation of *arg‐ii* gene in mice decreases organ aging phenotypes (Huang et al., 2021; Xiong, Yepuri, Necetin, et al., 2017; Yepuri et al., 2012). In contrast to Arg‐II, Arg‐I could not be detected in the mouse lung by immunoblotting, implicating that Arg‐II is the predominant isoform in mouse aging lung. This conclusion is further confirmed by the fact that arginase activity is fully abolished in the *arg‐ii* ^*−/−*^ mice. It is to mention that a species difference in expression of arginase isoforms exists. In the rat lung, both Arg‐I and Arg‐II are expressed particularly in the bronchiolar epithelial cells (Choi et al., 2012). We also found that Arg‐I is detectable in rat lung (but not in mouse and human lung biopsies) (Figure S13), demonstrating that Arg‐II is the predominant isoform in mouse and human lungs.

Further analysis of cellular localization of Arg‐II in the aging lung of mice and also in human lung biopsies by co‐immunostaining of Arg‐II with specific cellular markers reveals that Arg‐II is substantially expressed in alveolar AT2 pneumocytes, ciliated epithelial cells, club cells, and fibroblasts. Suprisingly, vascular endothelial cells and smooth muscle cells do not express Arg‐II in aging lung (Figure S8a,b). These observations are supported by single cell RNA‐sequencing in human lung which show no Arg‐II expression in pulmanory vascular endothelial cells, but in other cell types including epithelial cells, fibroblasts, and immune cells (https://www.proteinatlas.org/ENSG00000081181‐ARG2/single+cell+type/lung). We also found that pro‐fibrotic TGF‐β1 is widely expressed in various pulmonary cell types, including Arg‐II‐positive AT2 cells, bronchiolar epithelial cells, and fibroblasts (Figures 3d,e, 5a, Figure S10), but not in vascular endothelial cells (Figure S8c), and only marginal TGF‐β1 is found in some vascular smooth muscle cells (Figure S8d). The results suggest that Arg‐II and TGFβ1 are mainly produced in epithelial cells and fibroblasts in the aging lung, although a role of Arg‐II‐positive immune cells could not be excluded. The fact that the pro‐fibrotic TGF‐β1 is significantly increased in aging lung at both mRNA and protein levels, which is abolished in *arg‐ii* ^*−/−*^, suggests that Arg‐II could regulate TGF‐β1 production either in cell autonomous manner or paracrine manner. Indeed, in cell culture models of A549 (a widely used alveolar AT2‐epithelial cell model) and NL20 bronchiolar epithelial cells, TGF‐β1 levels are increased under hypoxic conditions, which is accompanied with increased Arg‐II levels. The increased TGF‐β1 levels in the epithelial cells are inhibited in *arg‐ii* ^*−/−*^ mice, demonstrating a cell autonomous effect of Arg‐II in stimulation of TGF‐β1 in epithelial cells. Moreover, we also show that conditioned media from hypoxic AT2 and bronchiolar epithelial cells are able to stimulate TGF‐β1 levels in fibroblasts accompanied with increased expression of *α‐sma*, pro‐inflammatiery cytokines including *il‐1β*, *il‐6* and *tnfα*, and matrix accumulation. The results demonstrate a paracrine effect of epithelial cells on activation of fibroblasts in cell culture model. These stimulating effects of fibroblasts by hypoxic epithelial conditioned media are abolished by either IL‐1β receptor antagonist or by TGF‐β type 1 receptor blocker, demonstrating that epithelial cells under hypoxia release IL‐1β and TGF‐β1 mediating pro‐fibrotic effects. The paracrine release by epithelial cells are further confirmed by increased levels of IL‐1β and TGF‐β1 in the conditioned media from hypoxic epithelial cells. Importantly, the hypoxia‐induced release of IL‐1β and TGF‐β1 is abolished in *arg‐ii* ^*−/−*^ epithelial cells, demonstrating a crucial role of Arg‐II in production of the two cytokines in the epithelial cells and a crosstalk between epithelial cells and fibroblasts. Finally, the results that increased IL‐1β and TGF‐β1 in AT2 pneumocytes and epithelial cells either in old mice or in mice exposed to hypoxia is inhibited in *arg‐ii* ^*−/−*^ animals, reinforce our conclusion and may represent an explanantion of hypoxia‐associated lung fibrosis (Gille et al., 2018; Lee et al., 2019). It is interesting to note that *il‐1β* expression is higher in aging lung when compared to the young and inhibited in *arg‐ii* ^*−/−*^. We found that IL‐1β receptor antagonist prevents increased hydroxyproline content in fibroblasts stimulated by hypoxic epithelial cell conditioned medium to the same extent as TGF‐β1 receptor blocker. This suggests that IL‐1β is able to stimulate TGF‐β1 expression in fibroblasts. Indeed, we found that TGF‐β1 levels in fibroblasts are increased when the cells are stimulated by IL‐1β. A definite conclusion on the crosstalk between epithelial cells and fibroblasts shall be confirmed in the future by generating epithelial specific *arg‐ii* ^*−/−*^ mice. Finally, we also show a positive circuit among Arg‐II, TGF‐β1, and IL‐1β, since TGF‐β1 is able to stimulate Arg‐II protein levels (Figure S11) and IL‐1β also increases both TGF‐β1 and Arg‐II levels in the fibroblasts (Figure 6F). The interaction among these three factors and the paracrine mechanisms between epithelial cells and fibroblasts shall play an important role in age‐associated pulmonary fibrosis.

Surprisingly, the age‐associated increase in Arg‐II levels in lung is observed only in females but not in males. This sex‐specific pattern of Arg‐II expression in aging lung differs slightly from other organs, example, kidney that shows an age‐associated increase in Arg‐II in both male and female with more pronounced increase in females (Huang et al., 2021). This finding indicates a sex‐ and organ‐specific regulation of Arg‐II in aging. The underlying mechanisms warrants further investigation.

Another finding of our study is the sex‐related difference in pulmonary aging phenotypes. Our naturally aging mouse model shows a much higher pulmonary hydroxyproline content in males than in age‐matched females (Figure 2i and Figure S5h). Although Arg‐II levels are comparable in the young animals between males and females (Figure S2a,b) and there is an age‐associated increase of Arg‐II in the female but not in male mice, the findings do not exclude a role of Arg‐II in pulmonary aging in male mice, but rather implicate a more predominant role of Arg‐II in females during aging as compared to males. The fact that the male *arg‐ii* ^*−/−*^ mice reveal lower levels of macrophage markers F4/80, hydroxyproline content, p16^ink4^ as compared with *wt* control animals despite no age‐associated increase in Arg‐II, suggests that basal levels of Arg‐II indeed play a role in pulmonary fibrosis in males. In addition, the results also suggest that other age‐associated mechanisms which are directly or indirectly linked to basal levels of Arg‐II must be involved in lung aging in males. The observation that there is an increase in TGF‐β1 level without changes in Arg‐II level in males, implicates a primary role of TGF‐β1 in collagen deposition in aged male mice. Whether the gender‐associated difference in Arg‐II expression with aging lung is also true for humans warrants further investigation.

Aging is associated with functional decline of the lung, to which it is partly contributed by a general lung fibrosis that causes increase in lung stiffness (Schneider et al., 2021). The increased hydroxyproline content is a marker for matrix deposition and reflects collagen content in aging lung (Kliment et al., 2011). The decreased hydroxyproline content in aging lung of *arg‐ii* ^*−/−*^ mice demonstrates a pro‐fibrotic effects of Arg‐II, which could be due to either stimulation of collagen synthesis and/or inhibition of collagen degradation. This aspect remains to be investigated. It is of note that during natural aging process in this mouse model, we see only few fibrotic foci in the old WT mice (Figure S3). It would be interesting to investigate in future whether Arg‐II also plays a role in other more severe pulmonary fibrosis models. It is to mention that our mouse model may not fully reflect human lung aging, in which complex interactions of genetic, epigenetic, environmental factors, diseases, and life style occur. However, chronic inflammation and fibrosis, although relatively mild in the mouse model, are still the characteristics of aging lung. Inflammation and fibrosis are distinct processes, but the two processes are highly intertwined each other, contributing to organ/tissue damage including lung. Understanding molecular and cellular mechanisms of inflammation and fibrosis in aging lung will provide novel insight or hints in aging‐associated pulmonary damage and how aging may contribute to various acute or chronic pulmonary diseases. Finally, the function of Arg‐II in club cells remains elusive and requires further investigation.

In conclusion, elevated Arg‐II in the lung promotes age‐related pulmonary inflammation and fibrosis in the cell autonomous and paracrine manner involving interaction between epithelial cells and fibroblasts, whereby IL‐1β and TGF‐β1 play a crucial role (Figure 6g). Our findings have potential clinical implications. First, Arg‐II but not Arg‐I (an enzyme involved in hepatic urea cycle) plays an important role in pulmonary inflammaging and fibrosis. Development of specific Arg‐II inhibitors is expected to have great potential to treat lung inflammaging and pulmonary fibrosis disease, particularly in women without risk of liver damage; second, given a key role of chronic inflammation in pulmonary fibrosis (Heukels et al., 2019; Kunzi et al., 2021) and that increased Arg‐II promotes inflammaging and fibrosis in other organs in aging (Huang et al., 2021; Yepuri et al., 2012), Arg‐II could be a therapeutic target in treatment of other organ fibrosis inaging, which may result in lifespan extension.

## 4 METHODS

### 4.1 Reagent and materials

Reagents were purchased from the following sources: TGF‐β receptor 1 inhibitor SB431542 (S1067, Selleckchem, Houston, USA), IL‐1 receptor antagonist IL‐1ra (280‐RA) and human IL‐1β (201‐LB/CF) from R&D systems (Minnesota, USA), Wheat Germ Agglutinin–Alexa Fluor 488 (W11261) from Invitrogen (Lucerne, Switzerland). All antibodies are presented in Table S1.

### 4.2 Animals and human samples

Wild type (*wt*) and *arg‐ii* knockout (*arg‐ii* ^*−/−*^) mice were kindly provided by Dr. William O'Brien (Shi et al., 2001) and back crossed to C57BL/6 J for more than 10 generations. Genotypes of mice were confirmed by polymerase chain reaction (PCR) as previously described. Offspring of *wt* and *arg‐ii* ^*−/−*^ mice were generated by interbred from hetero/hetero cross. Experimental work with animals was approved by the Ethical Committee of the Veterinary Office of Fribourg Switzerland (2020‐01‐FR) and performed in compliance with guidelines on animal experimentation at our institution.

Immunofluorescence staining was performed on human normal lung tissue fixed in formalin and embedded in paraffin (FFPE samples). Ethics approval was obtained in written form from the Ethics Committee of Northwestern and Central Switzerland (Project‐ID 2016‐01499). The study was conducted according to the principles expressed in the Declaration of Helsinki.

### 4.3 Mouse experiments of intermittent hypoxia (IH)

Female *wt* and *arg‐ii* ^*−/−*^ mice at the age of 5 months were randomly allocated into two groups that were exposed to normoxia (21% O_2_) or intermittent hypoxia (IH, 1.5 min at 8% O_2_ followed by 2.5 min of 20% O_2_ for 8 h per day during the light cycle from 9:00 AM to 17:00 AM) in the control cabinet or IH cabinet, respectively, of an Intermittent Hypoxic (IH)‐System for in Vivo Rapid Cycling (7,800,200, Coy Laboratory, Grass Lake, MI 49240, USA). Control animals were exposed to similar air–air cycles (compressed air) in order to be subject to equivalent levels of noise and air turbulences related to the gas circulation of the IH group (O_2_‐N_2_ cycles). And 21 days after exposure to either normoxia or IH, the mice were anesthetized with isoflurane and sacrificed by exsanguination. Right lung was snap frozen in liquid nitrogen and kept at −80°C until use. The left lung was cut transversely, fixed with 4% paraformaldehyde (pH 7.0), and then embedded in paraffin for immunofluorescence staining experiments.

### 4.4 Cell culture

A549 cells (the human lung alveolar epithelial cell line), NL‐20 cells (the human bronchial epithelial cell line), and MRC5 (the human lung fibroblast cell line) cells were purchased from ATCC and maintained in culture under standard conditions at 37°C and 5% CO_2_. Please see details in Supplementary Methods.

### 4.5 Generation of *arg‐ii* knockout cell lines using CRISP/Cas9 technologies

Please see details in Supplementary Methods.

### 4.6 Crosstalk between epithelial cells and fibroblasts

For these experiments, cells were seeded in six‐well plates with a density of 2 × 10^5^ cells per well. Before experiments, the cells were serum starved for 24 h. To collect the conditioned medium (CM) from epithelial cells, *wt* and *arg‐ii* ^*−/−*^ A549, *wt* and *arg‐ii* ^*−/−*^ NL‐20 cells were exposed to normoxia (21% O_2_) or hypoxia condition (1% O_2_) for 72 h. The CM collected from the A549 and NL‐20 cells (referred to as CM‐A549 and CM‐NL‐20, respectively) were then filtered and transferred ot MRC5 fibroblasts for 96 hours. To study effects of TGF‐β1 and/or IL‐1β in epithelial CM on MRC5 activation, MRC5 cells were pre‐treated either with TGF‐β receptor 1 inhibitor SB431542 (10 μmol/L) or IL‐1 receptor antagonist IL‐1ra (100 ng/mL) or the combination of both inhibitors before incubation with the CM. To confirm a role of IL‐1β on fibroblast activation, MRC5 cells were stimulated by human IL‐1β with different concentrations (1.25–5 ng/mL) for 48 hours. TGF‐β1 levels were analyzed by immunoblotting.

### 4.7 Immunoblotting

Lung tissue or cell lysate preparation, SDS‐PAGE and immunoblotting, antibody incubation, and signal detection were performed as described previously (Ming et al., 2012). Please see details in Supplementary Methods.

### 4.8 Arginase activity assay

Arginase activity assay was performed by colorimetric determination of urea formed from L‐arginine as previously described (Yepuri et al., 2012). Please see details in Supplementary Methods.

### 4.9 Real‐time quantitative RT‐PCR

mRNA expression was measured by two‐step quantitative Real Time‐PCR as described previously (Ming et al., 2012). All the RT‐PCR primer sequences are shown in the Table S2. Please see details in Supplementary Methods.

### 4.10 Immunofluorescence staining

Please see details in Supplementary Methods.

### 4.11 Masson's trichrome staining

The left mice lungs section (5 μm) was subjected to Masson's trichrome (ab150686, Abcam, Cambridge, UK) staining according to the manufacturer's instructions (Chen et al., 2017; Landini et al., 2020).

### 4.12 Hydroxyproline colorimetric assay

Collagen production was investigated by determination of hydroxyproline levels in right lung tissue or in cell homogenates using the Hydroxyproline Assay kit (MAK008, Sigma, Buchs, Switzerland) according to the manufacturer's instructions.

### 4.13 Elisa

TGF‐β1 and IL‐1β concentrations in the conditioned medium from human epithelial cells were measured by ELISA kits (human TGF‐β1, 88‐8350‐22, ThermoFisher, Waltham, Massachusetts, USA; human IL‐1β, 437,004, BioLegend,San Diego, USA) according to the manufacturer's instructions. In addition, blood was collected from mouse anaesthetized with 5% isoflurane in oxygen and maintained at 1.5% isoflurane during the procedure. Blood was taken with 30G insulin syringe (B. Braun; Melsungen; Germany) from jugular veins. After collection, blood was allowed to coagulate in a tube with gel clot activator (Microvette 500 Z‐Gel; SARSTED AG; Nümbrecht; Germany) for 15 min followed by centrifugation for 5 min at 10,000 × *g* to separate the serum. Serum was kept at −80 degree till TGFβ1 measurement using the ELISA kit as mentioned above.

### 4.14 Statistics

Data are presented as mean ± SD. Data distribution is determined by Kolmogorov–Smirnov test and statistical analysis for normally distributed values was performed with Student's unpaired t‐test or analysis of variance (ANOVA) with Bonferroni post hoc test. For non‐normally distributed values, Mann–Whitney test or the Kruskal–Wallis test was used. Differences in mean values were considered significant at a two‐tailed *p* ≤ 0.05.

## AUTHOR CONTRIBUTIONS

CZ, DMP, YY, GA conducted in vitro and in vivo experiments with mouse models, analyzed data, and intepreted results. SvG and KDM provided human biopsies and aided in interpreting results. ZY and X‐FM initiated, designed the project, provided financial support for the study, aided in analyzing results and drafted the manuscript. All the authors conbributed to editing the manuscript.

## FUNDING INFORMATION

This work was supported by the Swiss National Science Foundation (31003A_179261/1) and Swiss Heart Foundation (FF21021). CZ is a recipient of China Scholarship Council (CSC) Stipend. The laboratory of SvG is supported by grants from the Swiss National Science Foundation (310030_184757, 310030E_205559), and Swiss Cancer League/Swiss Cancer Research (KFS‐4958‐02‐2020).

## CONFLICT OF INTEREST STATEMENT

The authors have declared no conflict of interest.

## Supporting information


Data S1.

Table S1.
**Table S2**.

## Data Availability

All the data supporting the findings are available from corresponding author upon reasonable request.
