# Supplementary material for: Role of pulmonary epithelial arginase‐II in activation of fibroblasts and lung inflammaging
Source: Aging Cell. 2023 Feb 15;22(4):e13790. doi: 10.1111/acel.13790 (PMC10086530; doi:10.1111/acel.13790)
Supplement: Supplementary file 1 — Data S1. [file ACEL-22-e13790-s001.zip › ACEL_13790_Suppl.Figures-R1.pptx]

## Slide 1
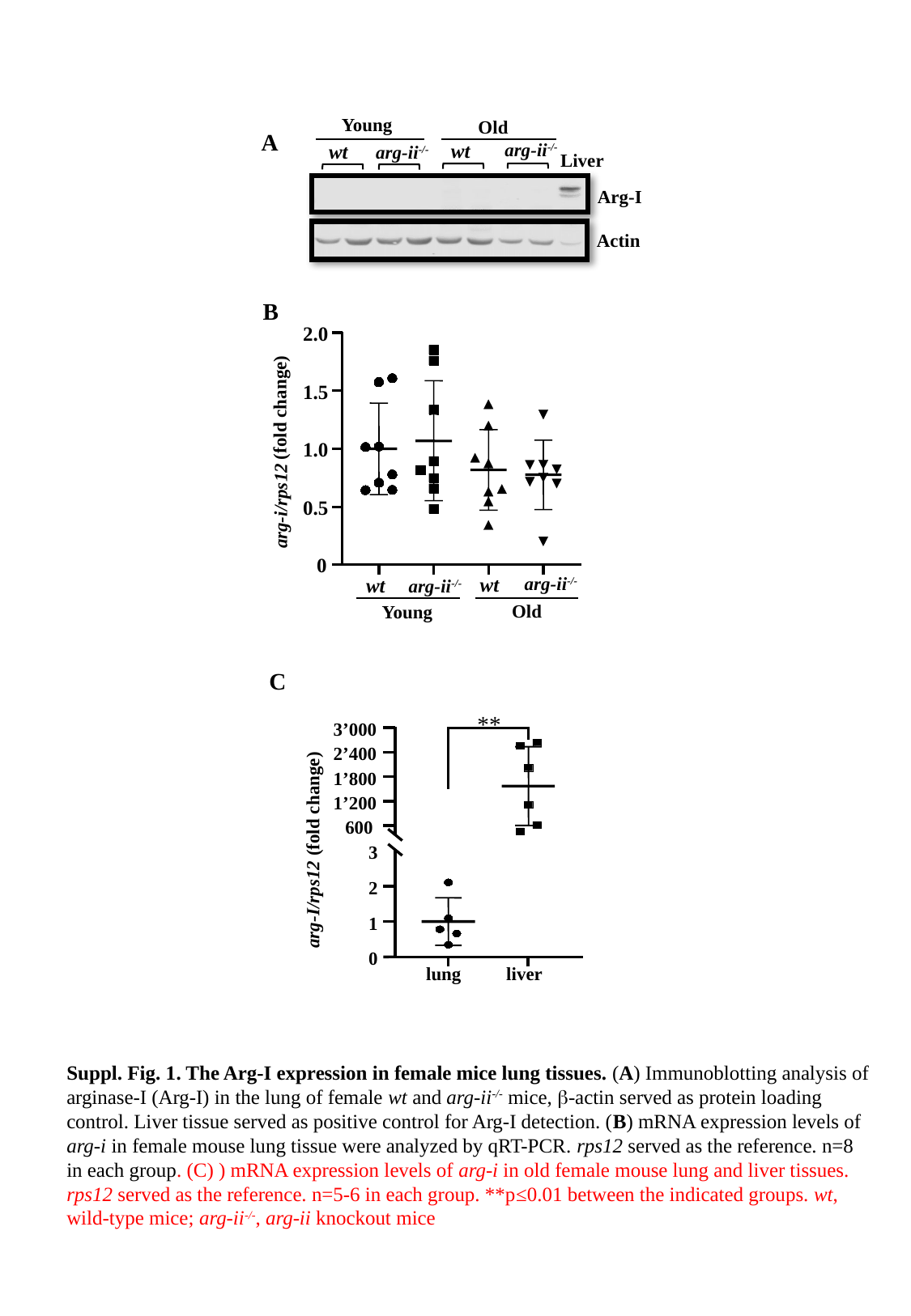

Young
Old
A
wt
arg-ii-/-
wt
arg-ii-/-
Liver
Arg-I
Actin
B
2.0
1.5
(fold change)
1.0
arg-i/rps12
0.5
0
wt
arg-ii-/-
wt
arg-ii-/-
Old
Young
C
**
3’000
2’400
1’800
1’200
600
arg-I/rps12 (fold change)
3
2
1
0
lung
liver
Suppl. Fig. 1. The Arg-I expression in female mice lung tissues. (A) Immunoblotting analysis of arginase-I (Arg-I) in the lung of female wt and arg-ii-/- mice, b-actin served as protein loading control. Liver tissue served as positive control for Arg-I detection. (B) mRNA expression levels of arg-i in female mouse lung tissue were analyzed by qRT-PCR. rps12 served as the reference. n=8 in each group. (C) ) mRNA expression levels of arg-i in old female mouse lung and liver tissues. rps12 served as the reference. n=5-6 in each group. **p≤0.01 between the indicated groups. wt, wild-type mice; arg-ii-/-, arg-ii knockout mice

## Slide 2
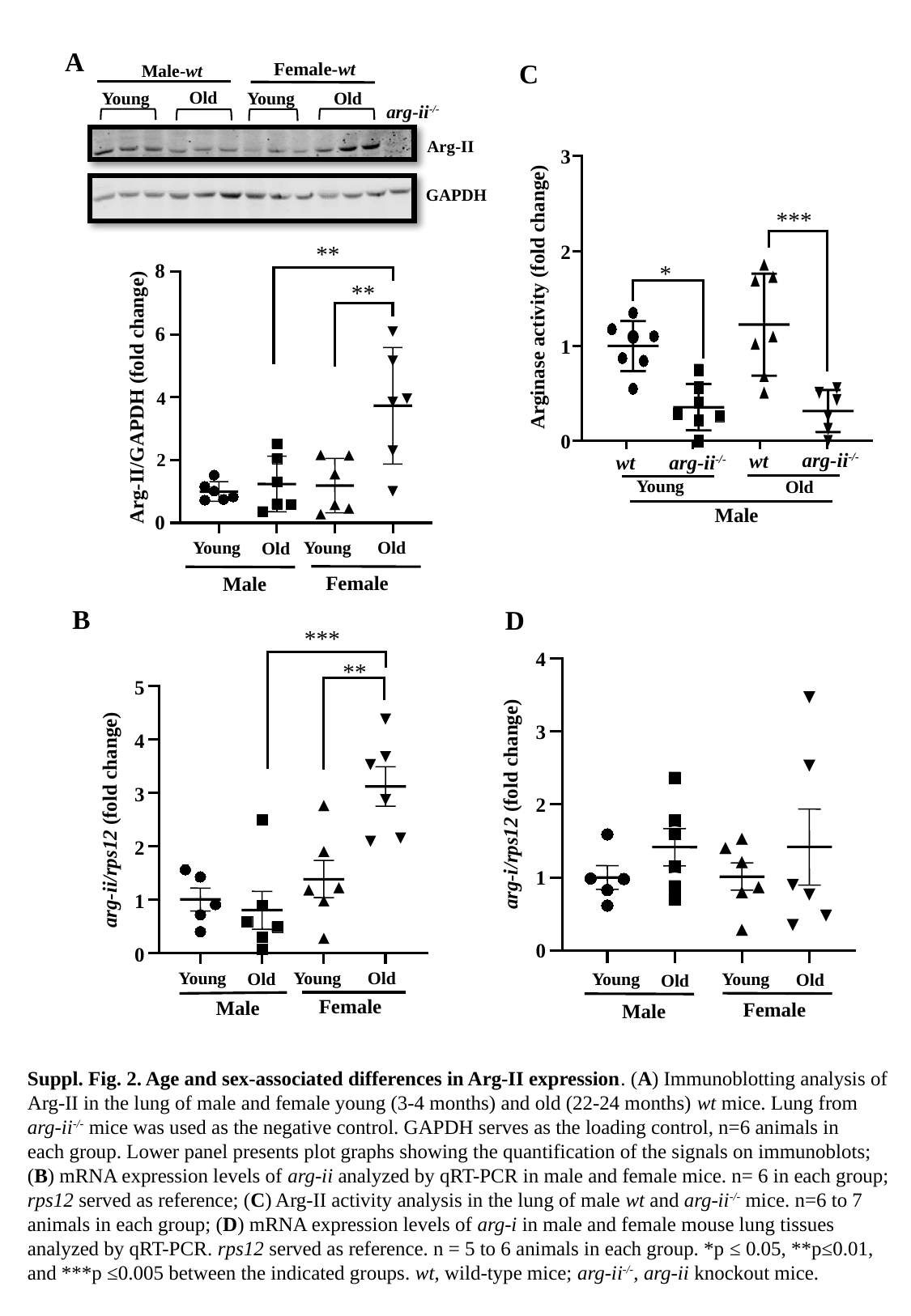

A
Female-wt
C
Male-wt
Old
Old
Young
Young
arg-ii-/-
Arg-II
3
***
2
*
Arginase activity (fold change)
1
0
arg-ii-/-
wt
wt
arg-ii-/-
Young
Old
Male
GAPDH
**
8
**
6
Arg-II/GAPDH (fold change)
4
2
0
Young
Young
Old
Old
Female
Male
B
D
***
4
**
5
3
4
3
2
arg-i/rps12 (fold change)
arg-ii/rps12 (fold change)
2
1
1
0
0
Young
 Young
 Old
Old
Young
Young
Old
Old
Female
Male
Female
Male
Suppl. Fig. 2. Age and sex-associated differences in Arg-II expression. (A) Immunoblotting analysis of
Arg-II in the lung of male and female young (3-4 months) and old (22-24 months) wt mice. Lung from
arg-ii-/- mice was used as the negative control. GAPDH serves as the loading control, n=6 animals in
each group. Lower panel presents plot graphs showing the quantification of the signals on immunoblots;
(B) mRNA expression levels of arg-ii analyzed by qRT-PCR in male and female mice. n= 6 in each group;
rps12 served as reference; (C) Arg-II activity analysis in the lung of male wt and arg-ii-/- mice. n=6 to 7
animals in each group; (D) mRNA expression levels of arg-i in male and female mouse lung tissues
analyzed by qRT-PCR. rps12 served as reference. n = 5 to 6 animals in each group. *p ≤ 0.05, **p≤0.01,
and ***p ≤0.005 between the indicated groups. wt, wild-type mice; arg-ii-/-, arg-ii knockout mice.

## Slide 3
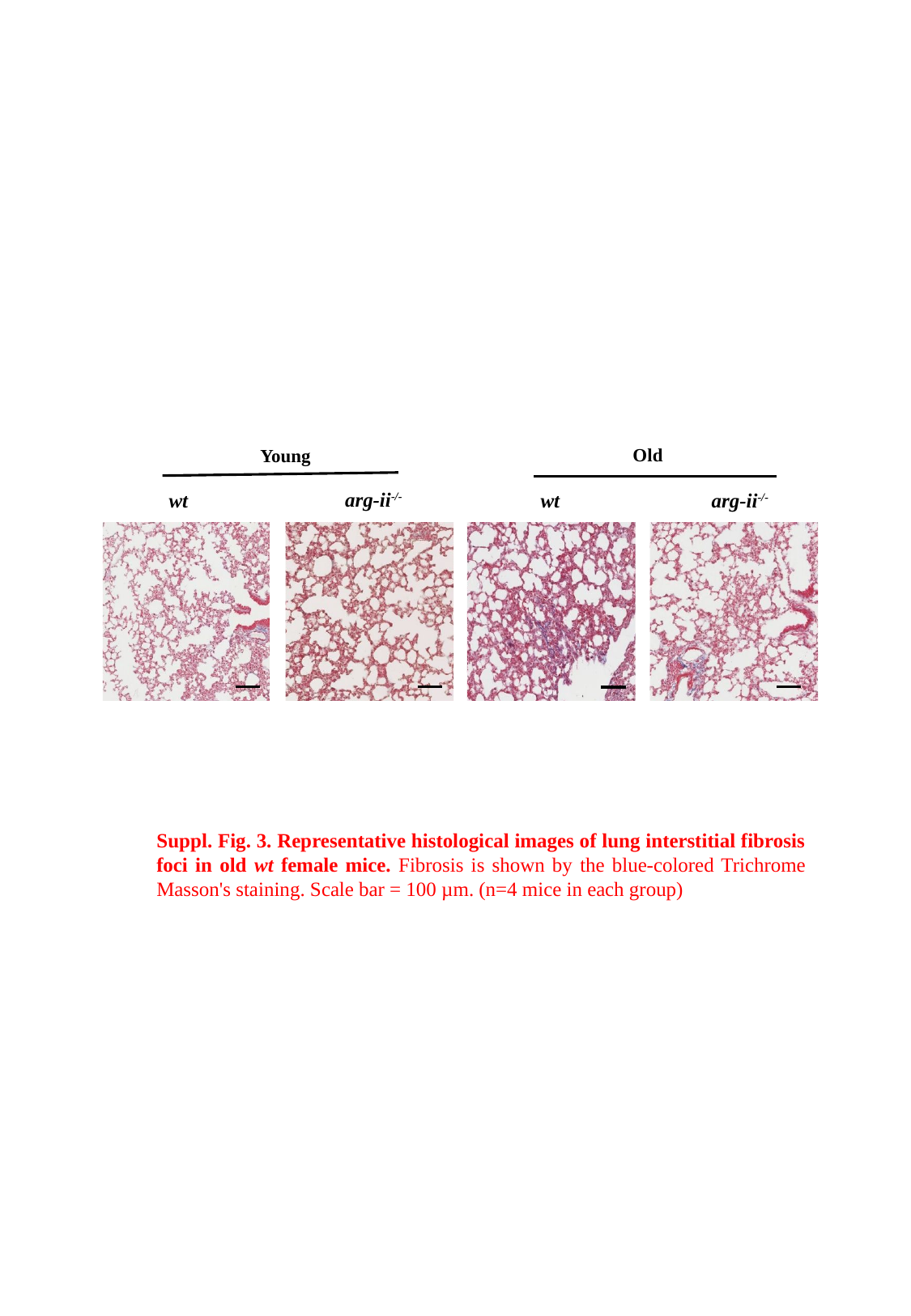

Old
Young
arg-ii-/-
arg-ii-/-
wt
wt
Suppl. Fig. 3. Representative histological images of lung interstitial fibrosis foci in old wt female mice. Fibrosis is shown by the blue-colored Trichrome Masson's staining. Scale bar = 100 µm. (n=4 mice in each group)

## Slide 4
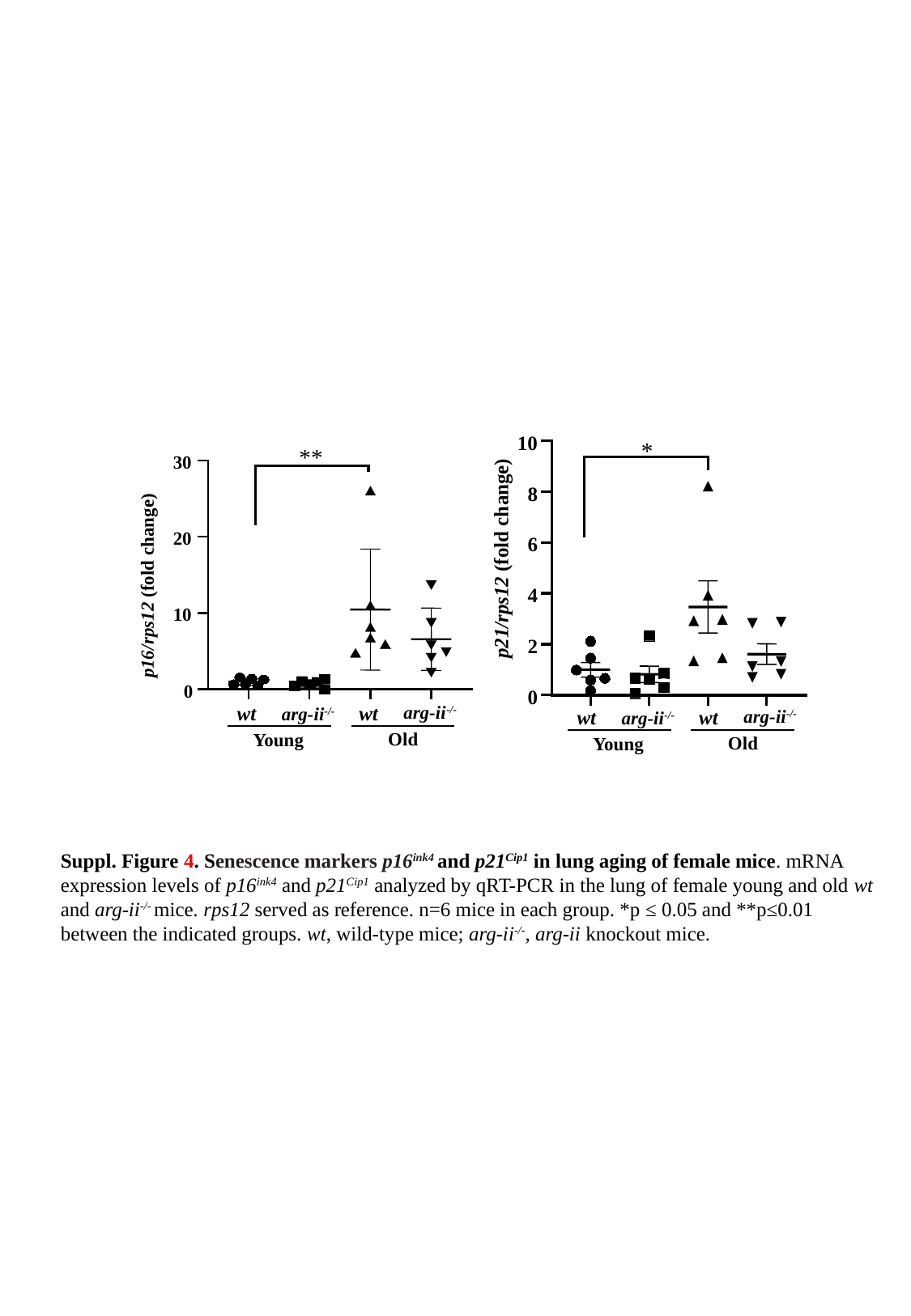

10
*
8
6
p21/rps12 (fold change)
4
2
0
**
30
20
p16/rps12 (fold change)
10
0
wt
arg-ii-/-
wt
arg-ii-/-
Old
Young
wt
arg-ii-/-
wt
arg-ii-/-
Old
Young
Suppl. Figure 4. Senescence markers p16ink4 and p21Cip1 in lung aging of female mice. mRNA expression levels of p16ink4 and p21Cip1 analyzed by qRT-PCR in the lung of female young and old wt and arg-ii-/- mice. rps12 served as reference. n=6 mice in each group. *p ≤ 0.05 and **p≤0.01 between the indicated groups. wt, wild-type mice; arg-ii-/-, arg-ii knockout mice.

## Slide 5
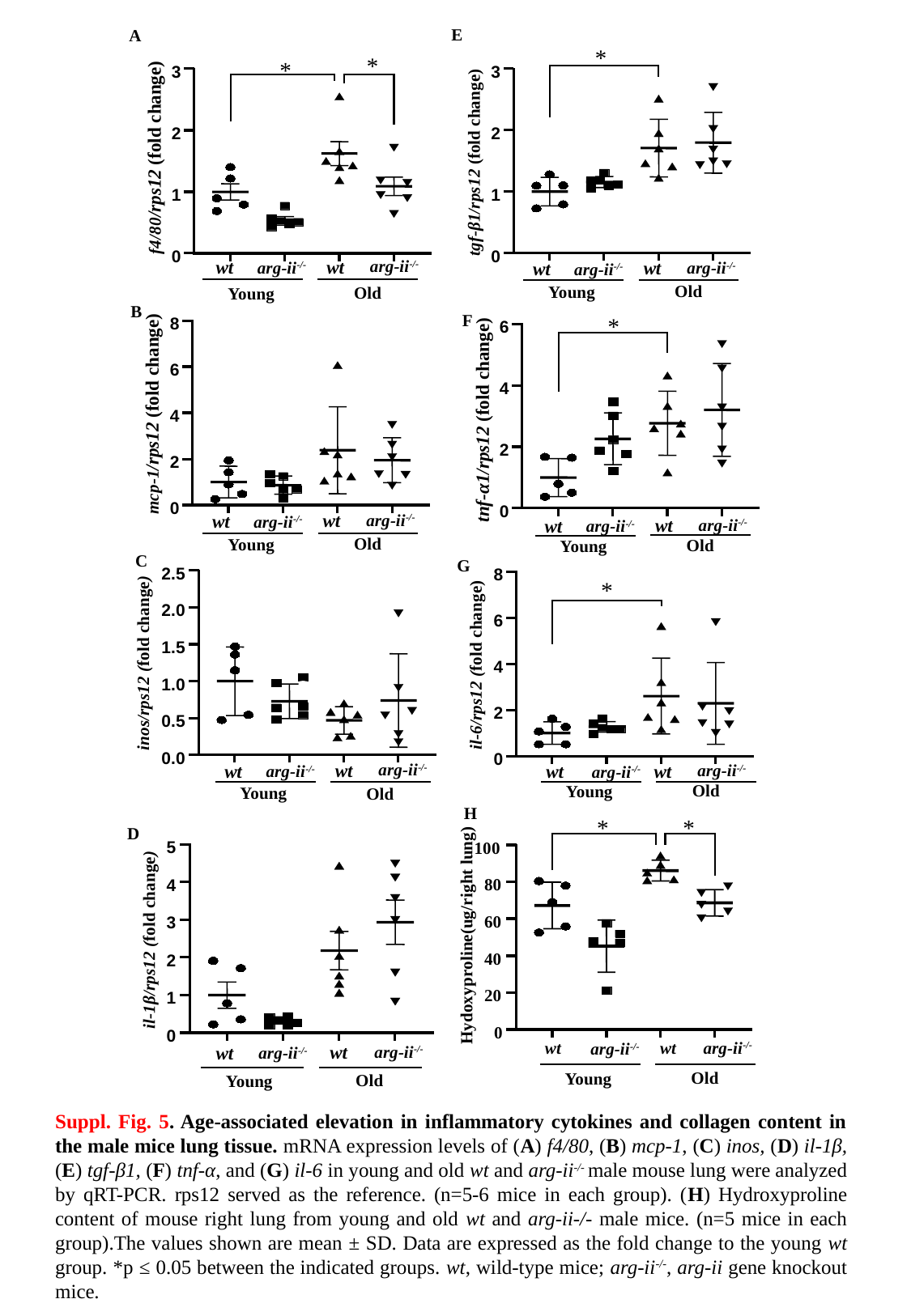

E
3
2
tgf-β1/rps12 (fold change)
1
0
A
3
2
f4/80/rps12 (fold change)
1
0
wt
arg-ii-/-
wt
arg-ii-/-
Old
Young
*
*
*
wt
arg-ii-/-
wt
arg-ii-/-
Old
Young
B
8
6
mcp-1/rps12 (fold change)
4
2
0
wt
arg-ii-/-
wt
arg-ii-/-
Old
Young
*
F
6
4
tnf-α1/rps12 (fold change)
2
0
wt
arg-ii-/-
wt
arg-ii-/-
Old
Young
C
G
2.5
8
*
2.0
6
1.5
inos/rps12 (fold change)
il-6/rps12 (fold change)
4
1.0
2
0.5
0.0
0
wt
arg-ii-/-
wt
arg-ii-/-
Young
Old
wt
arg-ii-/-
wt
arg-ii-/-
Old
Young
H
*
*
D
5
100
80
4
60
3
Hydoxyproline(ug/right lung)
il-1β/rps12 (fold change)
40
2
20
1
0
0
wt
arg-ii-/-
wt
arg-ii-/-
Old
Young
wt
arg-ii-/-
wt
arg-ii-/-
Old
Young
Suppl. Fig. 5. Age-associated elevation in inflammatory cytokines and collagen content in the male mice lung tissue. mRNA expression levels of (A) f4/80, (B) mcp-1, (C) inos, (D) il-1β, (E) tgf-β1, (F) tnf-α, and (G) il-6 in young and old wt and arg-ii-/- male mouse lung were analyzed by qRT-PCR. rps12 served as the reference. (n=5-6 mice in each group). (H) Hydroxyproline content of mouse right lung from young and old wt and arg-ii-/- male mice. (n=5 mice in each group).The values shown are mean ± SD. Data are expressed as the fold change to the young wt group. *p ≤ 0.05 between the indicated groups. wt, wild-type mice; arg-ii-/-, arg-ii gene knockout mice.

## Slide 6
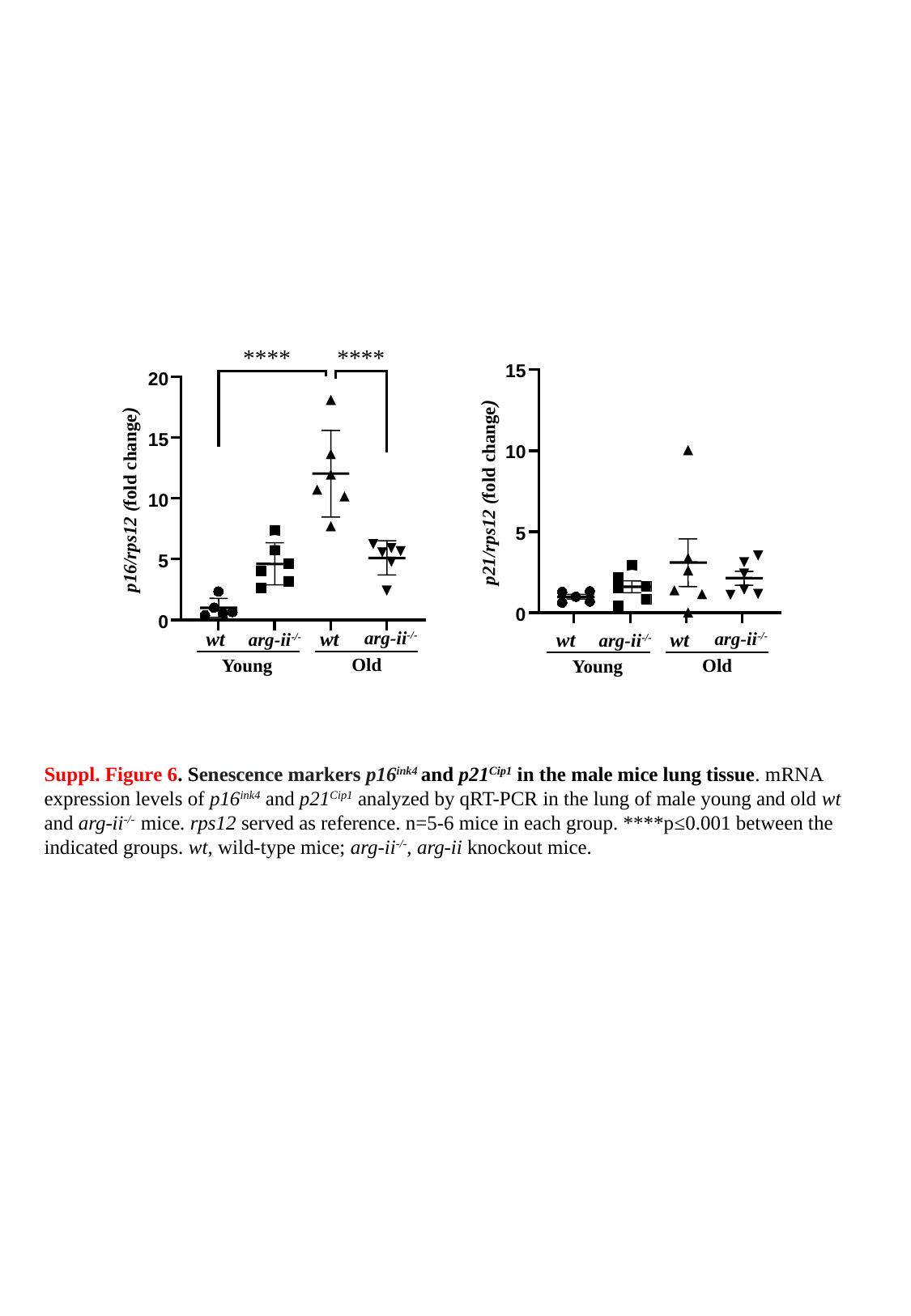

****
****
20
15
p16/rps12 (fold change)
10
5
0
wt
arg-ii-/-
wt
arg-ii-/-
Old
Young
15
10
p21/rps12 (fold change)
5
0
wt
arg-ii-/-
wt
arg-ii-/-
Old
Young
Suppl. Figure 6. Senescence markers p16ink4 and p21Cip1 in the male mice lung tissue. mRNA expression levels of p16ink4 and p21Cip1 analyzed by qRT-PCR in the lung of male young and old wt and arg-ii-/- mice. rps12 served as reference. n=5-6 mice in each group. ****p≤0.001 between the indicated groups. wt, wild-type mice; arg-ii-/-, arg-ii knockout mice.

## Slide 7
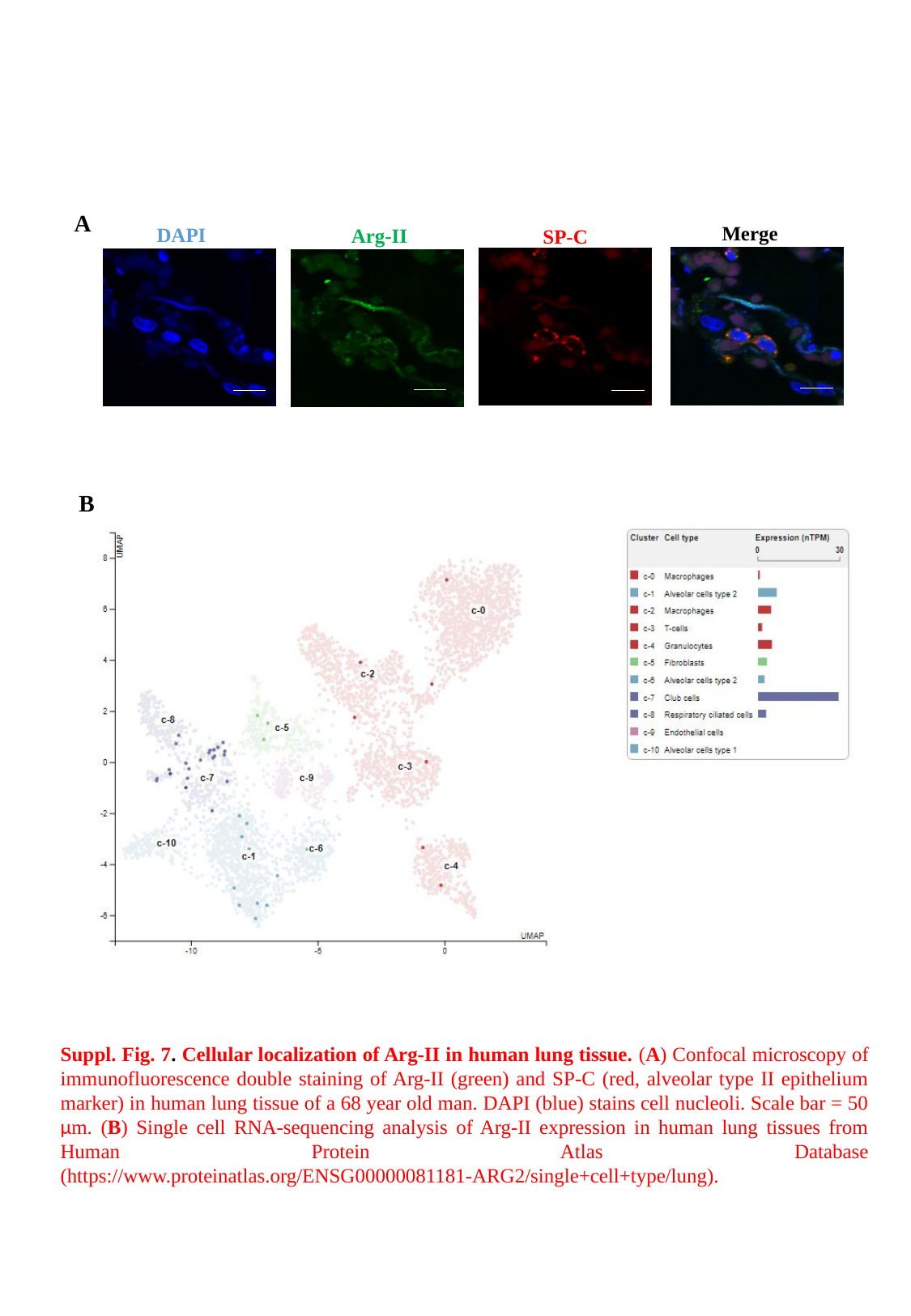

A
Merge
DAPI
Arg-II
SP-C
B
Suppl. Fig. 7. Cellular localization of Arg-II in human lung tissue. (A) Confocal microscopy of immunofluorescence double staining of Arg-II (green) and SP-C (red, alveolar type II epithelium marker) in human lung tissue of a 68 year old man. DAPI (blue) stains cell nucleoli. Scale bar = 50 µm. (B) Single cell RNA-sequencing analysis of Arg-II expression in human lung tissues from Human Protein Atlas Database (https://www.proteinatlas.org/ENSG00000081181-ARG2/single+cell+type/lung).

## Slide 8
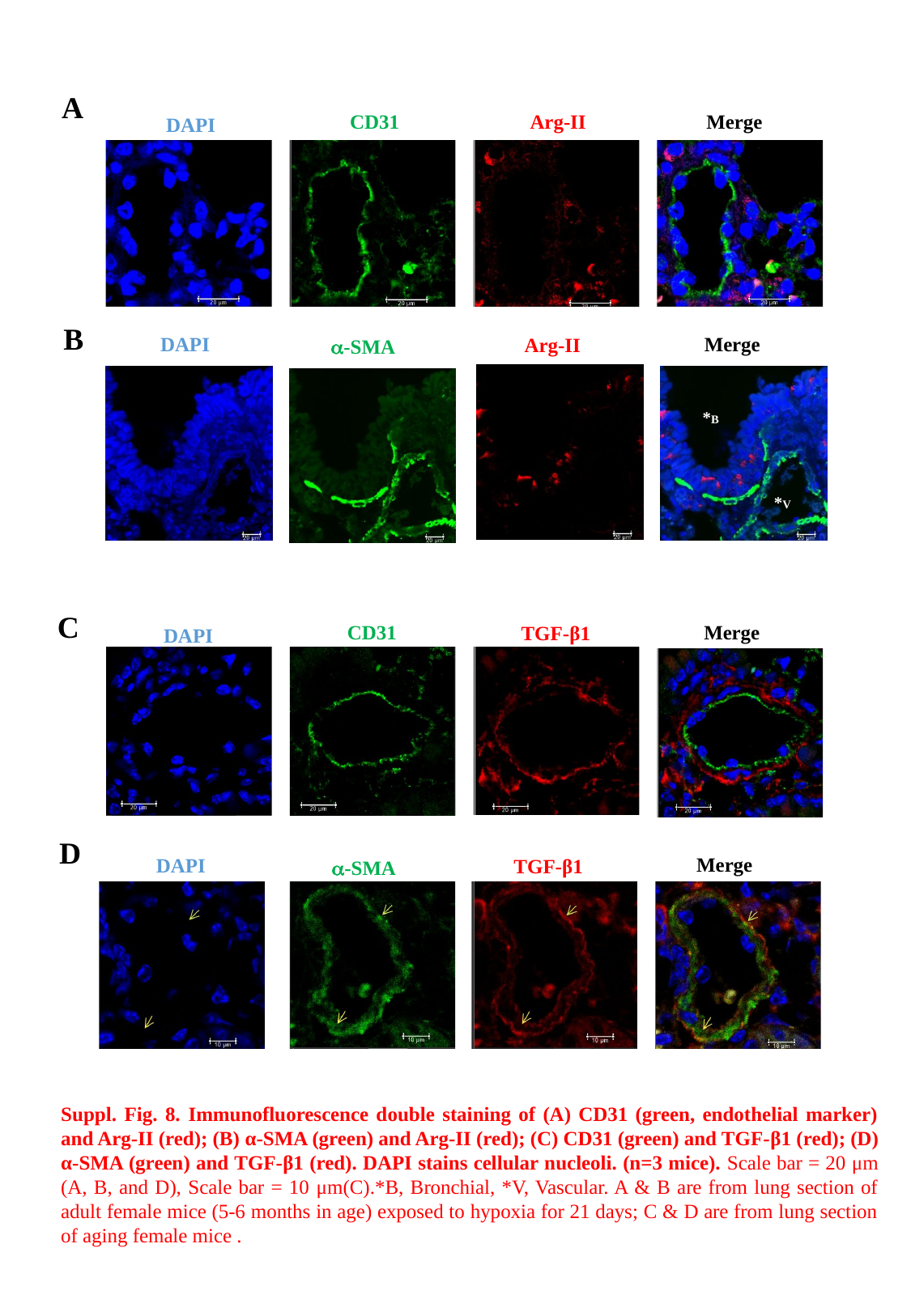

A
CD31
Merge
Arg-II
DAPI
B
Merge
DAPI
Arg-II
a-SMA
*B
*V
C
CD31
Merge
TGF-β1
DAPI
D
Merge
DAPI
TGF-β1
a-SMA
Suppl. Fig. 8. Immunofluorescence double staining of (A) CD31 (green, endothelial marker) and Arg-II (red); (B) α-SMA (green) and Arg-II (red); (C) CD31 (green) and TGF-β1 (red); (D) α-SMA (green) and TGF-β1 (red). DAPI stains cellular nucleoli. (n=3 mice). Scale bar = 20 μm (A, B, and D), Scale bar = 10 μm(C).*B, Bronchial, *V, Vascular. A & B are from lung section of adult female mice (5-6 months in age) exposed to hypoxia for 21 days; C & D are from lung section of aging female mice .

## Slide 9
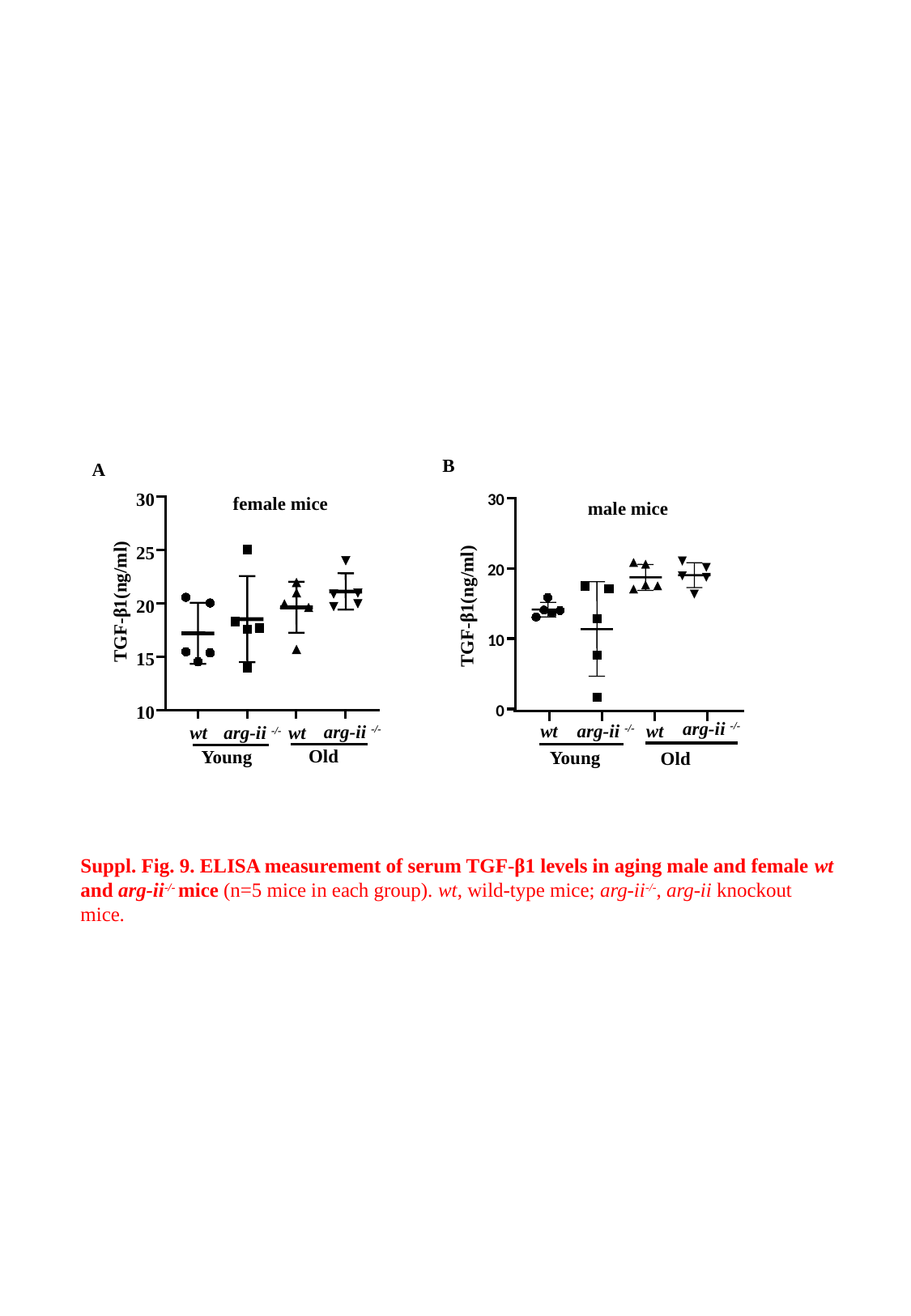

B
A
30
30
female mice
male mice
25
20
TGF-β1(ng/ml)
20
TGF-β1(ng/ml)
10
15
0
10
arg-ii -/-
wt
arg-ii -/-
wt
arg-ii -/-
wt
arg-ii -/-
wt
Old
Young
Young
Old
Suppl. Fig. 9. ELISA measurement of serum TGF-β1 levels in aging male and female wt and arg-ii-/- mice (n=5 mice in each group). wt, wild-type mice; arg-ii-/-, arg-ii knockout mice.

## Slide 10
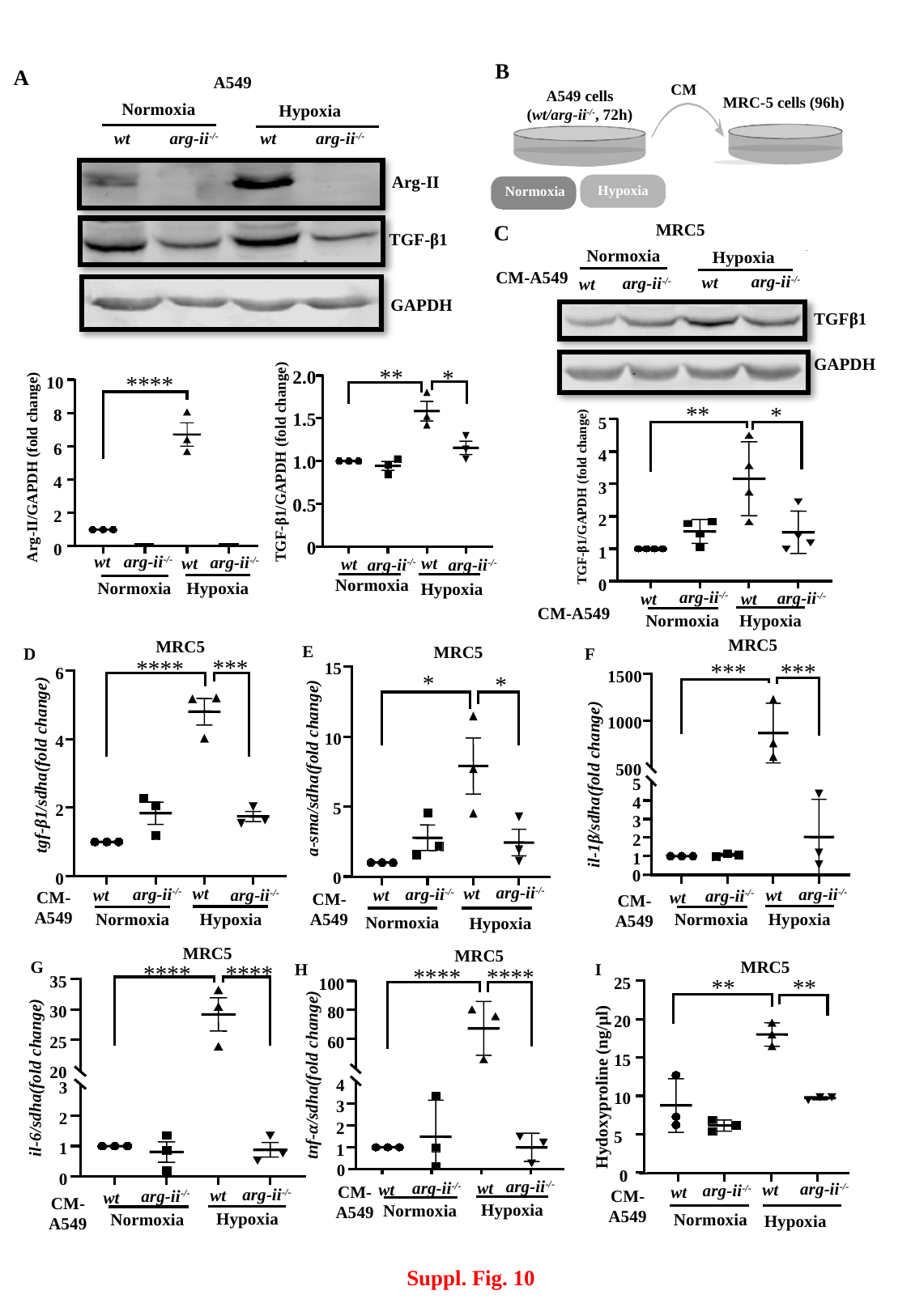

B
CM
A549 cells
(wt/arg-ii-/-, 72h)
Hypoxia
Normoxia
MRC-5 cells (96h)
A
A549
wt
wt
arg-ii-/-
arg-ii-/-
Normoxia
Hypoxia
Arg-II
TGF-β1
GAPDH
MRC5
C
Normoxia
Hypoxia
CM-A549
arg-ii-/-
wt
arg-ii-/-
wt
TGFβ1
GAPDH
**
*
2.0
1.5
1.0
TGF-β1/GAPDH (fold change)
0.5
 0
wt
arg-ii-/-
arg-ii-/-
wt
Normoxia
Hypoxia
****
10
8
6
Arg-II/GAPDH (fold change)
4
2
0
wt
arg-ii-/-
arg-ii-/-
wt
Hypoxia
Normoxia
**
*
5
4
3
TGF-β1/GAPDH (fold change)
2
1
0
arg-ii-/-
arg-ii-/-
wt
wt
Hypoxia
Normoxia
CM-A549
MRC5
F
***
***
1500
1000
500
5
il-1β/sdha(fold change)
4
3
2
1
0
arg-ii-/-
wt
arg-ii-/-
wt
Hypoxia
Normoxia
CM-
A549
MRC5
D
***
****
6
4
tgf-β1/sdha(fold change)
2
0
wt
arg-ii-/-
arg-ii-/-
wt
CM-
A549
Normoxia
Hypoxia
E
MRC5
15
*
*
10
a-sma/sdha(fold change)
5
0
arg-ii-/-
wt
arg-ii-/-
wt
CM-
A549
Normoxia
Hypoxia
MRC5
G
****
****
35
30
25
20
il-6/sdha(fold change)
3
2
1
0
arg-ii-/-
wt
arg-ii-/-
wt
Hypoxia
Normoxia
CM-
A549
MRC5
H
****
****
100
80
60
tnf-α/sdha(fold change)
4
3
2
1
0
MRC5
I
**
**
25
20
15
Hydoxyproline (ng/μl)
10
5
 0
arg-ii-/-
wt
arg-ii-/-
wt
CM-
A549
Normoxia
Hypoxia
arg-ii-/-
wt
arg-ii-/-
wt
Hypoxia
Normoxia
CM-
A549
Suppl. Fig. 10

## Slide 11
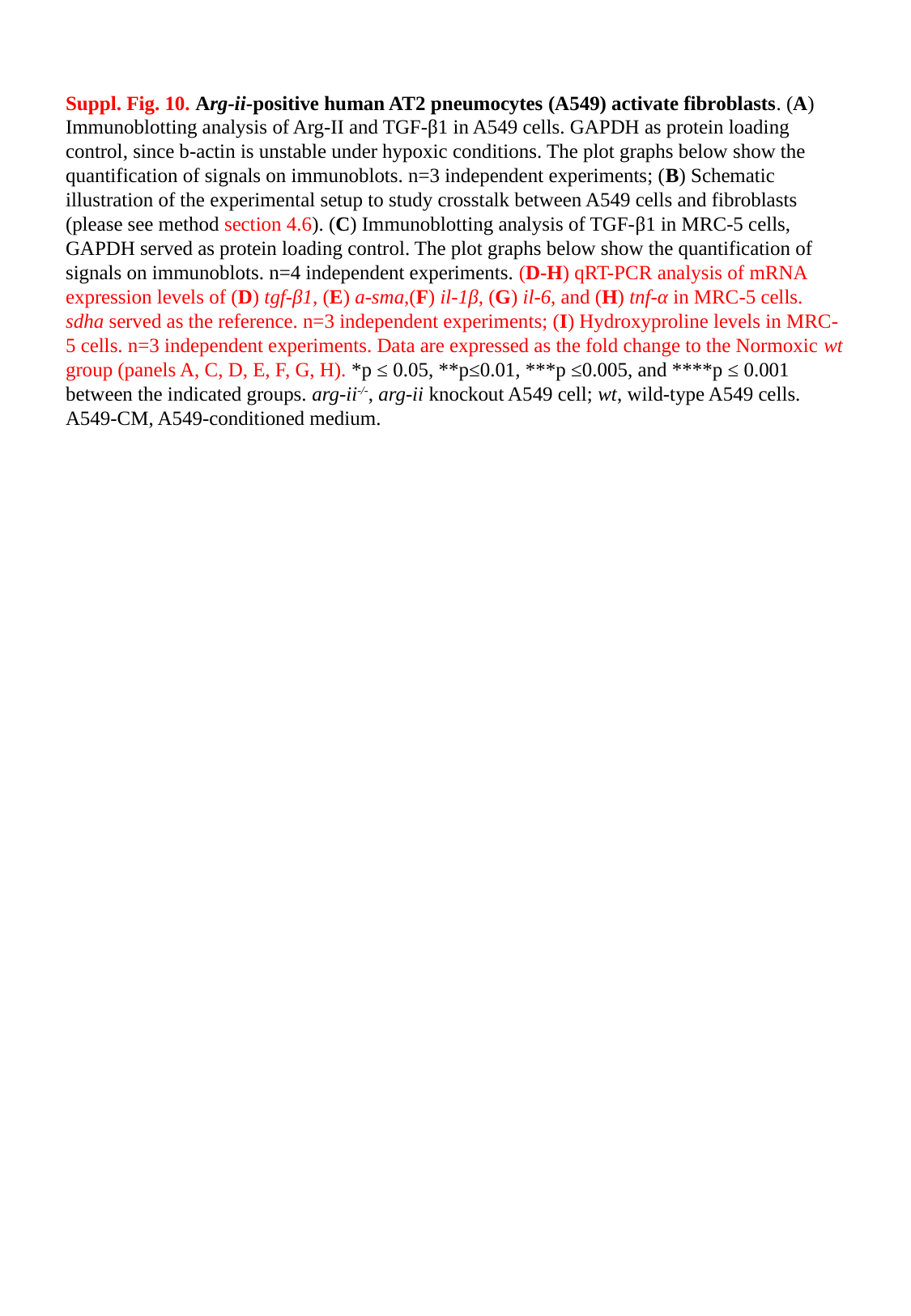

Suppl. Fig. 10. Arg-ii-positive human AT2 pneumocytes (A549) activate fibroblasts. (A) Immunoblotting analysis of Arg-II and TGF-β1 in A549 cells. GAPDH as protein loading control, since b-actin is unstable under hypoxic conditions. The plot graphs below show the quantification of signals on immunoblots. n=3 independent experiments; (B) Schematic illustration of the experimental setup to study crosstalk between A549 cells and fibroblasts (please see method section 4.6). (C) Immunoblotting analysis of TGF-β1 in MRC-5 cells, GAPDH served as protein loading control. The plot graphs below show the quantification of signals on immunoblots. n=4 independent experiments. (D-H) qRT-PCR analysis of mRNA expression levels of (D) tgf-β1, (E) a-sma,(F) il-1β, (G) il-6, and (H) tnf-α in MRC-5 cells. sdha served as the reference. n=3 independent experiments; (I) Hydroxyproline levels in MRC-5 cells. n=3 independent experiments. Data are expressed as the fold change to the Normoxic wt group (panels A, C, D, E, F, G, H). *p ≤ 0.05, **p≤0.01, ***p ≤0.005, and ****p ≤ 0.001 between the indicated groups. arg-ii-/-, arg-ii knockout A549 cell; wt, wild-type A549 cells. A549-CM, A549-conditioned medium.

## Slide 12
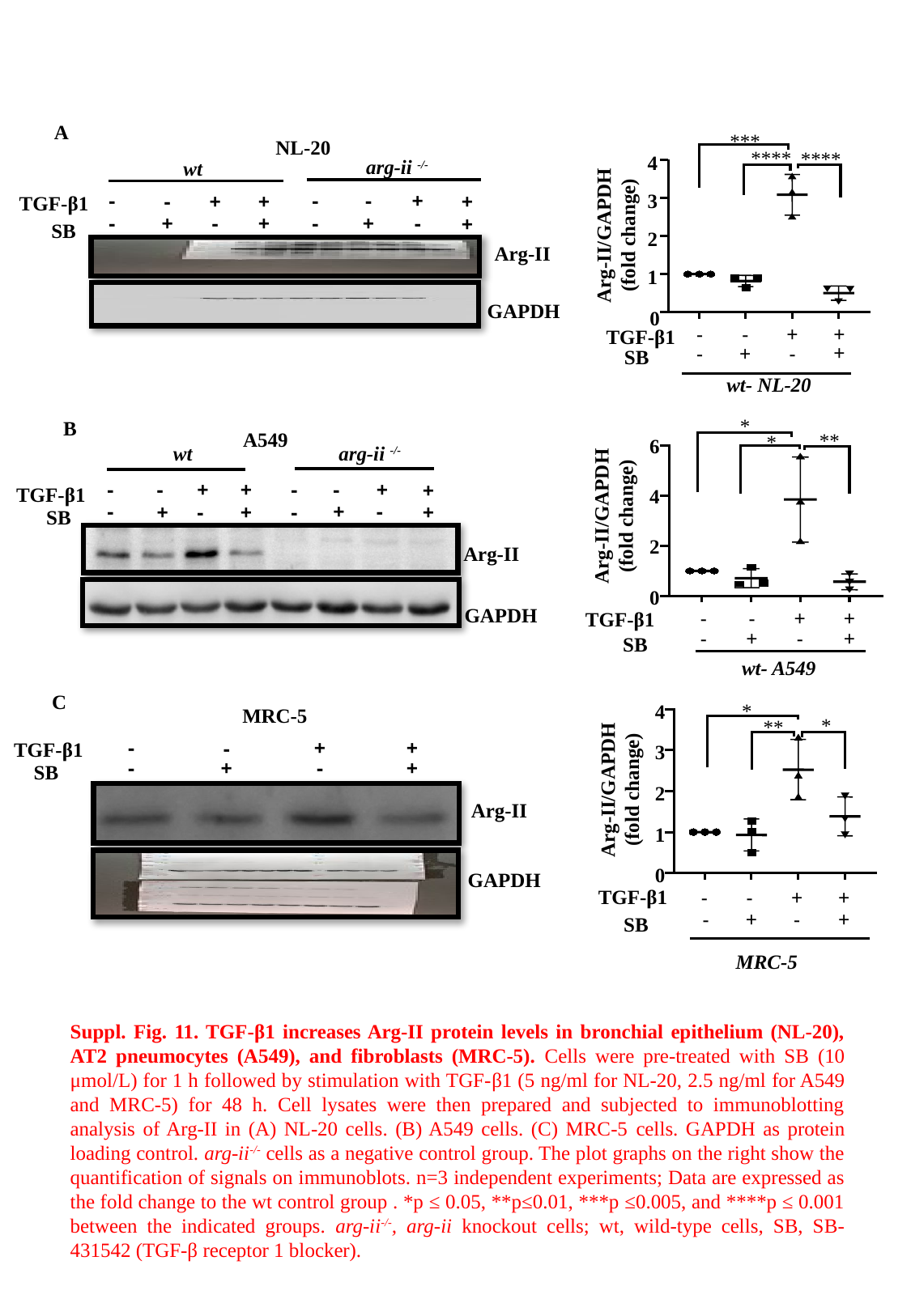

A
NL-20
arg-ii -/-
wt
-
-
-
+
-
+
+
+
TGF-β1
+
-
-
+
+
-
-
+
SB
Arg-II
GAPDH
***
****
****
4
3
Arg-II/GAPDH
(fold change)
2
1
0
-
+
+
-
+
-
-
+
SB
wt- NL-20
TGF-β1
B
A549
arg-ii -/-
wt
-
-
-
+
-
+
+
+
TGF-β1
+
-
-
+
+
-
-
+
SB
Arg-II
GAPDH
*
**
*
6
4
Arg-II/GAPDH
(fold change)
2
0
+
-
+
-
TGF-β1
+
-
-
+
SB
wt- A549
C
MRC-5
-
+
+
-
TGF-β1
+
-
-
+
SB
Arg-II
GAPDH
*
4
*
**
3
Arg-II/GAPDH
(fold change)
2
1
0
TGF-β1
-
+
+
-
+
-
-
+
SB
MRC-5
Suppl. Fig. 11. TGF-β1 increases Arg-II protein levels in bronchial epithelium (NL-20), AT2 pneumocytes (A549), and fibroblasts (MRC-5). Cells were pre-treated with SB (10 μmol/L) for 1 h followed by stimulation with TGF-β1 (5 ng/ml for NL-20, 2.5 ng/ml for A549 and MRC-5) for 48 h. Cell lysates were then prepared and subjected to immunoblotting analysis of Arg-II in (A) NL-20 cells. (B) A549 cells. (C) MRC-5 cells. GAPDH as protein loading control. arg-ii-/- cells as a negative control group. The plot graphs on the right show the quantification of signals on immunoblots. n=3 independent experiments; Data are expressed as the fold change to the wt control group . *p ≤ 0.05, **p≤0.01, ***p ≤0.005, and ****p ≤ 0.001 between the indicated groups. arg-ii-/-, arg-ii knockout cells; wt, wild-type cells, SB, SB-431542 (TGF-β receptor 1 blocker).

## Slide 13
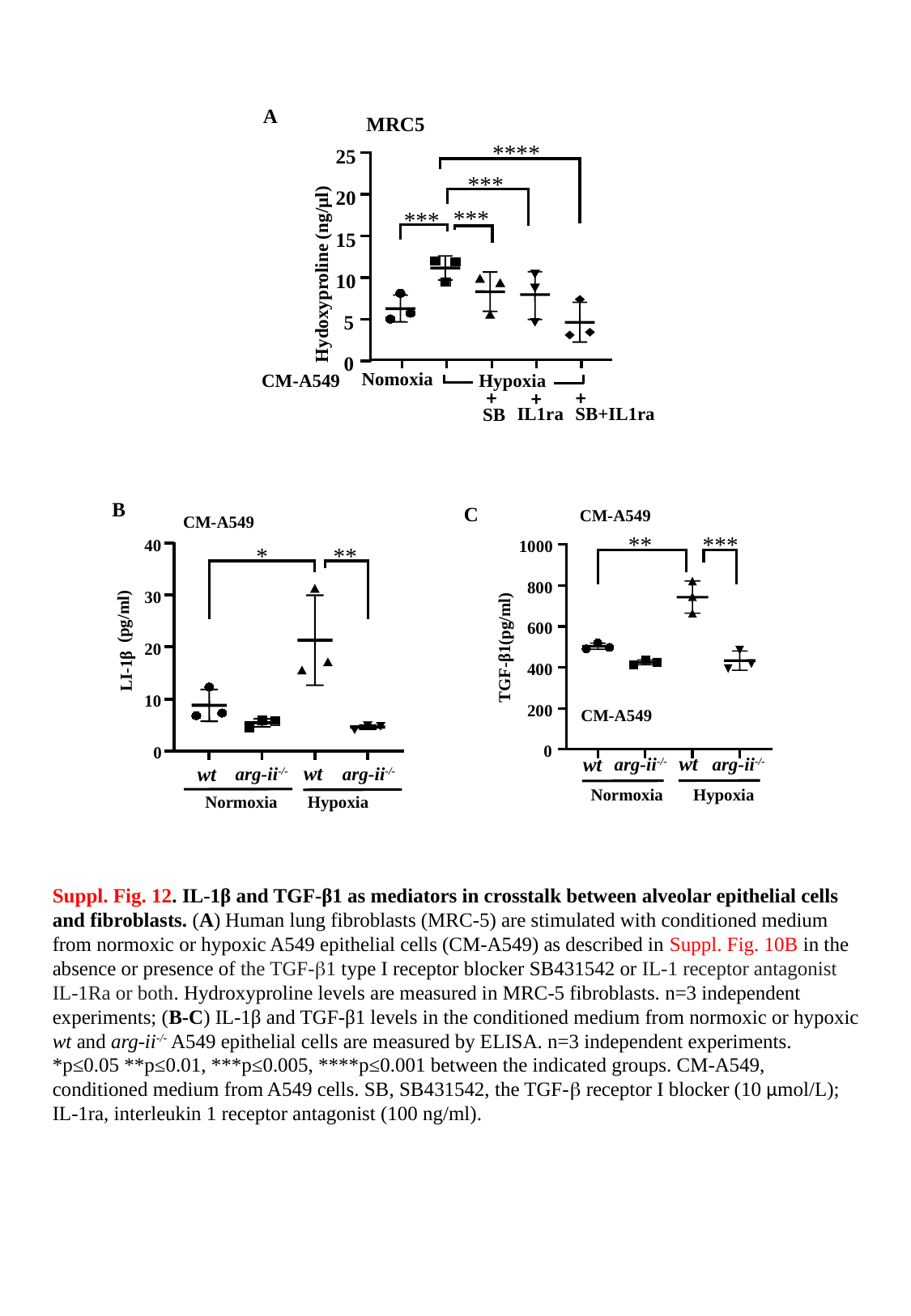

A
MRC5
****
25
***
20
***
***
15
Hydoxyproline (ng/μl)
10
5
0
Nomoxia
Hypoxia
+
+
+
SB+IL1ra
IL1ra
SB
CM-A549
B
CM-A549
wt
wt
arg-ii-/-
arg-ii-/-
Hypoxia
Normoxia
40
*
**
30
(pg/ml)
LI-1β
20
10
0
C
CM-A549
**
***
1000
800
600
TGF-β1(pg/ml)
400
CM-A549
200
0
wt
wt
arg-ii-/-
arg-ii-/-
Hypoxia
Normoxia
Suppl. Fig. 12. IL-1β and TGF-β1 as mediators in crosstalk between alveolar epithelial cells and fibroblasts. (A) Human lung fibroblasts (MRC-5) are stimulated with conditioned medium from normoxic or hypoxic A549 epithelial cells (CM-A549) as described in Suppl. Fig. 10B in the absence or presence of the TGF-b1 type I receptor blocker SB431542 or IL-1 receptor antagonist IL-1Ra or both. Hydroxyproline levels are measured in MRC-5 fibroblasts. n=3 independent experiments; (B-C) IL-1β and TGF-β1 levels in the conditioned medium from normoxic or hypoxic wt and arg-ii-/- A549 epithelial cells are measured by ELISA. n=3 independent experiments. *p≤0.05 **p≤0.01, ***p≤0.005, ****p≤0.001 between the indicated groups. CM-A549, conditioned medium from A549 cells. SB, SB431542, the TGF-b receptor I blocker (10 µmol/L); IL-1ra, interleukin 1 receptor antagonist (100 ng/ml).

## Slide 14
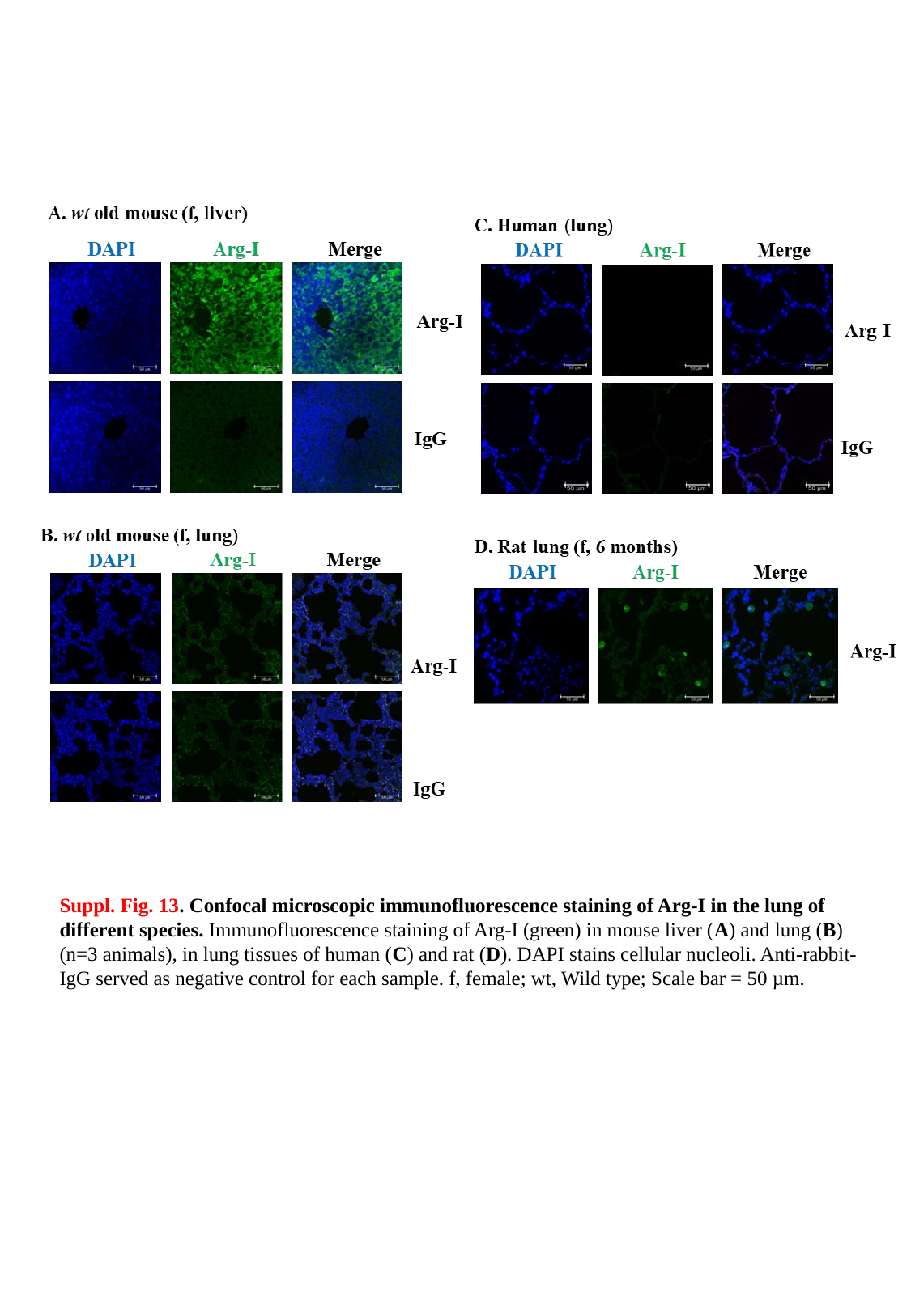

Suppl. Fig. 13. Confocal microscopic immunofluorescence staining of Arg-I in the lung of different species. Immunofluorescence staining of Arg-I (green) in mouse liver (A) and lung (B) (n=3 animals), in lung tissues of human (C) and rat (D). DAPI stains cellular nucleoli. Anti-rabbit-IgG served as negative control for each sample. f, female; wt, Wild type; Scale bar = 50 µm.
