# Supplementary material for: Role of pulmonary epithelial arginase‐II in activation of fibroblasts and lung inflammaging
Source: Aging Cell. 2023 Feb 15;22(4):e13790. doi: 10.1111/acel.13790 (PMC10086530; doi:10.1111/acel.13790)
Supplement: Supplementary file 1 — Data S1. [file ACEL-22-e13790-s001.zip › ACEL_13790_Supplementary Methods.docx]

*(November 9^th^, 2022)*

*Research article*

*Aging Cell*

*ACE-22-0514R1*

**Role of pulmonary epithelial arginase-II in activation of fibroblasts and lung inflammaging**

Cui Zhu^1^, Duilio M. Potenza^1^, Yang Yang^1^, Guillaume Ajalbert^1^, Kirsten D. Mertz^2^,

Stephan von Gunten^3^, Xiu-Fen Ming^1^*, Zhihong Yang^1^*

^1^Laboratory of Cardiovascular and Aging Research, Department of Endocrinology, Metabolism, and Cardiovascular System, Faculty of Science and Medicine, University of Fribourg, Switzerland; ^2^Institute for Pathology, Cantonal Hospital Baselland, Liestal, Switzerland, ^3^Institute of Pharmacology, Faculty of Medicine, University of Bern, Switzerland

*Corresponding authors

**Running title: Arginase-II and lung inflammaging**

**Email addresses of authors :**

Cui Zhu: [cui.zhu@unifr.ch](mailto:cui.zhu@unifr.ch); Duilio M. Potenza: [duilio.potenza@unifr.ch](mailto:duilio.potenza@unifr.ch);

Yang Yang: [yang.yang@unifr.ch](mailto:yang.yang@unifr.ch); Guillaume Ajalbert: [guillaume.ajalbert@unifr.ch](mailto:guillaume.ajalbert@unifr.ch);

Kirsten D. Mertz: [Kirsten.Mertz@ksbl.ch](mailto:Kirsten.Mertz@ksbl.ch);

Stephan von Gunten: [stephan.vongunten@pki.unibe.ch](mailto:stephan.vongunten@pki.unibe.ch);

Xiu-Fen Ming: [xiu-fen.ming@unifr.ch](mailto:xiu-fen.ming@unifr.ch); Zhihong Yang: [zhihong.yang@unifr.ch](mailto:zhihong.yang@unifr.ch)

**Statements relating to relevant ethics:** Experimental work with animals was approved by the Ethical Committee of the Veterinary Office of Fribourg Switzerland (2020-01-FR) and performed in compliance with guidelines on animal experimentation at our institution. Experiments with human lung tissues were approved by the Ethics Committee of Northwestern and Central Switzerland (Project-ID 2016-01499). The study was conducted according to the principles expressed in the Declaration of Helsinki.

**Supplemental Materials and Methods**.

**4.1 Reagent and materias**

Reagents were purchased from the following sources:Rabbit antibody against Arg-II (#55003) was from Cell Signaling Technology (Danvers, USA). Mouse-antibody against Arg-I (610708) was purchased from BD Transduction Laboratories (New Jersey, USA). Rabbit antibody against TGF-β1 (ab215715), IL-1β (ab9722) and mouse-antibody against a-SMA (ab7817) were obtained from Abcam (Cambridge, UK). Mouse-antibody against SP-C (sc-519029), CC-10 (sc-365992) and TGFβ1 (sc-130348) were from Santa Cruz (Nunningen, Switzerland). Mouse-antibody against FOXJ1 (14-9965-82) was purchased from Thermo Fisher Scientific (Waltham, Massachusetts, USA). Mouse-antibody against β-actin (A5441) and GAPDH (10R-G109A) were obtained from Sigma-Aldrich (Buchs, Switzerland). IRDye 800-conjugated affinity purified goat anti-rabbit IgG (9263221) was from BioConcept (Allschwil, Switzerland). Alexa fluor 680-conjugated goat anti-mouse IgG (A-21057) was purchased from Invitrogen (Lucerne, Switzerland). Alexa Fluor 488-conjugated goat anti-rabbit IgG (H+L) secondary Ab (A-11008), Alexa Fluor 594-conjugated goat anti-rabbit IgG (H+L) secondary Ab (A-11012), Alexa Fluor 488-conjugated goat anti-mouse IgG (H+L) secondary Ab (A-11001) and Alexa Fluor 568-conjugated goat anti-mouse IgG (H+L) secondary Ab (A-11031) were purchased from Thermo Fisher Scientific (Waltham, Massachusetts, USA). TGF-β receptor 1 inhibitor SB431542 (S1067) was obtained from Selleckchem (Houston, USA). IL-1 receptor antagonist IL-1ra (280-RA) and human IL-1β (201-LB/CF) were purchased from R&D systems (Minnesota, USA). Wheat Germ Agglutinin–Alexa Fluor 488 (W11261) was purchased from Invitrogen (Lucerne, Switzerland).

**4.2 Animals and human samples**

Wild type (*wt*) and *arg-ii* knockout (*arg-ii^−/−^*) mice were kindly provided by Dr. William O’Brien (Shi et al., 2001) and back crossed to C57BL/6 J for more than 10 generations. Genotypes of mice were confirmed by polymerase chain reaction (PCR) as previously described. Offspring of *wt* and *arg-ii^−/−^* mice were generated by interbred from hetero/hetero cross. Mice were housed at 23°C with a 12-h-light-dark cycle. Animals were fed a normal chow diet and had free access to water. Male and female mice at age of 3–4 months (young) or 22–24 months (old) were anesthetized with isoflurane and sacrificed by exsanguination. The right lung was snap frozen in liquid nitrogen and kept at −80 °C until use. The left lung was cut transversely, fixed with 4% paraformaldehyde (pH 7.0), and then embedded in paraffin for immunofluorescence staining experiments. Experimental work with animals was approved by the Ethical Committee of the Veterinary Office of Fribourg Switzerland (2020-01-FR) and performed in compliance with guidelines on animal experimentation at our institution.

Immunofluorescence staining was performed on human normal lung tissue fixed in formalin and embedded in paraffin (FFPE samples). Ethics approval was obtained in written form from the Ethics Committee of Northwestern and Central Switzerland (Project-ID 2016-01499). The study was conducted according to the principles expressed in the Declaration of Helsinki.

**4.3 Mouse experiments of intermittent hypoxia (IH)**

Female *wt* and *arg-ii^−/−^* mice at the age of 5 months were randomly allocated into two groups that were exposed to normoxia (21% O_2_) or intermittent hypoxia (IH, 1.5 min at 8% O_2_ followed by 2.5 min of 20% O_2_ for 8 hours per day during the light cycle from 9:00AM to 17:00AM) in the control cabinet or IH cabinet, respectively, of an Intermittent Hypoxic (IH)-System for in Vivo Rapid Cycling (7800200, Coy Laboratory, Grass Lake, MI 49240, USA). Control animals were exposed to similar air-air cycles (compressed air) in order to be subject to equivalent levels of noise and air turbulences related to the gas circulation of the IH group (O_2_-N_2_ cycles). 21 days after exposure to either normoxia or IH, the mice were anesthetized with isoflurane and sacrificed by exsanguination. Right lung was snap frozen in liquid nitrogen and kept at −80 °C until use. The left lung was cut transversely, fixed with 4% paraformaldehyde (pH 7.0), and then embedded in paraffin for immunofluorescence staining experiments.

**4.4 Cell culture**

A549 cells (the human lung alveolar epithelial cell line) were cultured in RPMI 1640 Medium (PAN Biotech,Germany) containing 10% fetal bovine serum (FBS; Gibco, Zug,Switzerland) and 1% streptomycin and penicillin. NL-20 cells (the human bronchial epithelial cell line) were cultured in Dulbecco's modified Eagle's medium (DMEM; Thermo Fisher Scientific, Waltham, Massachusetts, USA) supplemented with 0.005 mg/ml insulin, 10 ng/ml epidermal growth factor, 0.001 mg/ml transferrin, 500 ng/ml hydrocortisone, 4% FBS and 1% streptomycin and penicillin. MRC5 (the human lung fibroblast cell line) cells were cultured in DMEM containing 10% FBS and 1% streptomycin and penicillin. All these cells were purchased from ATCC and maintained in culture under standard conditions at 37℃ and 5% CO_2_. The culture media were changed every other day.

**4.5 Generation of *arg-ii* knockout cell lines using CRISP/Cas9 technologies**

sgRNA targeting human *arg-ii* (the top strand of the sgRNA that recognizes the target DNA region of human *arg-ii*: GGGACTAACCTATCGAGA was cloned into pSpCas9(BB)-2A-Puro (PX459) V2.0 (Plasmid #62988, addgene) to generate pSpCas9(BB)-2A-Puro (PX459)-U6/sgRNA-harg-ii. A549 and NL-20 cells were plated in 6-cm dish at a density of 1 × 10^6^ cells 24 h before transfection. Transfection of pSpCas9(BB)-2A-Puro (PX459)-U6/sgRNA-*harg-ii* was performed using Lipofectamine™ 3000 Transfection Reagent (L3000008, Invitrogen™) according to the manufacturer's protocol. Briefly, per 1 × 10^6^ cells, diluted plasmid DNA (5 µg, diluted with P3000™ Reagent) and diluted Lipofectamine™ 3000 Transfection Reagent were mixed at a 1:1 ratio and incubated at room temperature for 15 min. The DNA-lipid complex was then added to the cells. To select the sgRNA-positive cells, 48 h post transfection, cells were treated with puromycin (2.5 µg/ml) for 48 hours until all the control cells without transfection died. Puromycin-resistant cells were allowed to recover in medium without puromycin for 1 week before seeding single cells into 96-well plate by dilution method. Single clones were then expanded and screened for Arg-II by immunoblotting.

**4.6 Crosstalk between epithelial cells and fibroblasts**

For these experiments, cells were seeded in six-well plates with a density of 2 × 10^5^ cells per well. Before experiments, the cells were serum starved for 24 h. To collect the conditioned medium (CM) from epithelial cells, *wt* and *arg-ii^−/−^* A549, *wt* and *arg-ii^−/−^* NL-20 cells were exposed to normoxia (21% O_2_) or hypoxia condition (1% O_2_) for 72 hours. The CM collected from the A549 and NL-20 cells (referred to as CM-A549 and CM-NL-20, respectively) were then filtered and transferred ot MRC5 fibroblasts for 96 hours. To study effects of TGF-β1 and/or IL-1β in epithelial CM on MRC5 activation, MRC5 cells were pre-treated either with TGF-β receptor 1 inhibitor SB431542 (10 μmol/L) or IL-1 receptor antagonist IL-1ra (100 ng/ml) or the combination of both inhibitors before incubation with the CM. To confirm a role of IL-1β on fibroblast activation, MRC5 cells were stimulated by human IL-1β with different concentrations (1.25 to 5 ng/ml) for 48 hours. TGF-β1 levels were analyzed by immunoblotting.

**4.7 Immunoblotting**

Lung tissue or cell lysate preparation, SDS-PAGE and immunoblotting, antibody incubation, and signal detection were performed as described previously (Ming et al., 2012). β-actin was used as protein loading control, except for experiments with hypoxia, since β-actin was altered under hypoxic conditions. For the experiments with hypoxia, GAPDH was then used as protein loading control. To prepare lung homogenates, frozen lung tissues were crushed into the fine powder using a mortar and pestle in a liquid nitrogen bath on ice. A portion of the fine powder was then homogenized (XENOX-Motorhandstück MHX homogenizer) on ice in 150 µl of ice-cold lysis buffer with the following composition: 10 mmol/L Tris-HCl (pH 7.4), 0.4% Triton X-100, 10 μg/mL leupeptin, and 0.1 mmol/L phenylmethylsulfonyl fluoride (PMSF), protease inhibitor cocktail (B14002) and phosphatase inhibitor cocktail (B15002; Bio-tool). Homogenates were centrifuged (Sorvall Legend Micro 17R) at 13,800 × g for 15 min at 4 °C. Protein concentrations of the supernatant were then determined by Lowry method (500-0116, Bio-Rad). Equal amount of protein from each sample was heated at 95°C for 5 min in loading buffer and separated by SDS-PAGE electrophoresis. Proteins in the SDS-PAGE gel were then transferred to PVDF membranes which were blocked with PBS-Tween-20 supplemented with 5% skimmed milk. The membranes were then incubated with the corresponding primary antibody overnight at 4°C with gentle agitation. After washing with blocking buffer, the membranes were then incubated with corresponding anti-mouse (Alexa Fluor 680-conjugated) or anti-rabbit (IRDye 800-conjugated) secondary antibodies. Signals were visualized using the Odyssey Infrared Imaging System (LI-COR Biosciences) and quantified by NIH Image J 1.60 (US NIH). The antibodies used in this study are shown in the **Suppl. Table 1**.

**4.8 Arginase activity assay**

Arginase activity assay was performed by colorimetric determination of urea formed from L-arginine as previously described (Xiong et al., 2013). Briefly, the lung tissue lysates were prepared in lysis buffer containing 10 mmol/L Tris-HCl (pH 7.4), 0.4% Triton X-100, 10 μg/mL leupeptin, and 0.1 mmol/L phenylmethylsulfonyl fluoride (PMSF). Samples were centrifuged at 13’000 rpm at 4°C for 10 minutes, and the protein concentration of the supernatant was determined by the Lowry method (Bio-Rad). Arginase activity assay, 150 µg protein of the lung lysate in 50 µl lysis buffer was added to 50 µl of Tris-HCl (10 mmol/L [pH 7.4]) containing 5 mmol/L MnCl_2_. Arginase was then activated by heating the mixture at 56°C for 10 minutes. The hydrolysis reaction of L-arginine by arginase was conducted by incubating the mixture containing activated arginase with 100 µof L-arginine (100 mmol/L [pH 9.6]) at 37°C for 1 hour. For colorimetric determination of urea, 1 mL of chromogenic reagent consisting of 1 volume of 3% 2,3-butanedione monoxime and 29 volumes of the acid solution mixture (H_2_SO_4_:H_3_PO_4_:H_2_O 1:3:7) was added, and the mixture was then heated at 100°C for 30 minutes. After placing the samples in the dark for 10 minutes at room temperature, the urea concentration in the reaction was determined spectrophotometrically by the absorbance at 492 nm. The amount of urea produced was used as an index for arginase activity.

**4.9 Real-time quantitative RT-PCR**

mRNA expression of the inflammatory markers, *p16^ink4^, p21^cip1^, arg-ii, arg-i, α-sma* was measured by two-step quantitative Real Time-PCR as described previously (Ming et al., 2012). Ribosomal Protein S12 (*rps12*) was used as reference for mouse tissues and *sdha* was used as reference for human cells. Total RNA was extracted from mouise left lungs and MRC-5 cells with Trizol Reagent (TR-118, Molecular Research Center) following the manufacturer’s protocol. Real-time PCR reaction was performed with the GOTaq® qPCR Master Mix (A6001, Promega) and CFX96 Real-Time PCR Detection System (Bio-Rad). The mRNA expression levels of all genes were quantified using the standard curve method and were further normalized to the reference gene *rps12* or *sdha*. All the RT-PCR primer sequences are shown in the **Suppl. Table 2**.

**4.10 Immunofluorescence staining**

Mouse left lungs were isolated and fixed with 4% paraformaldehyde (pH 7.0) and embedded in paraffin. After deparaffinization in xylene (3 times,5 min for each), the sections were treated in ethanol (twice in 100% ethanol, twice in 95% ethanol, and once in 80%, 75%, 50% ethanol for 5 min, sequentially) followed by antigen retrieval (Tris- EDTA buffer, pH 9.0 for Arg-II, CC-10, SP-C, FOXJ1, TGF-β1, a-SMA; Citrate buffer, pH 6.0 for SP-C and IL-1β) in a pressure cooker. For co-immunofluorescence staining of Arg-II/SP-C, Arg-II/CC-10, Arg-II/FOXJ1, Arg-II/TGF-β1, TGF-β1/SP-C, TGF-β1/a-SMA, IL-1β/SP-C, primary antibodies of different species were used. For triple co-immunofluorescence staining of Arg-II/TGF-β1/PDGF-Rα, primary antibodies of different species were used. Co-staining of Arg-II with CC-10 and FOXJ1 respectively (see figure 3B and C), was followed by a staining with Wheat Germ Agglutinin (WGA)-Alexa Fluor 488 for visualizing cell membranes (10 μg/mL x 30 min). This technique was employed to define cell border and ensure co-localization of the proteins in the same cell. Transverse sections (5 μm) were blocked with mouse Ig blocking reagent (M.O.M, Vector laboratories, California,USA) for 3 h and then with PBS containing 1% BSA and 10% goat serum for 1 h. The sections were then incubated overnight at 4°C in a dark/humidified chamber with anti-rabbit Arg-II, TGF-β1, IL-1β, and, anti-mouse SP-C, CC-10, FOXJ1, TGF-β1, and α-SMA antibody, and subsequently incubated for 2 h with the following secondary antibodies: Alexa Fluor 488–conjugated goat anti-rabbit IgG (H + L) and Alexa Fluor 568-conjugated goat anti-mouse IgG (H + L), or Alexa Fluor 488–conjugated goat anti-mouse IgG (H + L) and Alexa Fluor 594-conjugated goat anti-rabbit IgG (H + L). All the sections were finally counterstained with 300 nmol/L DAPI for 5 min. Immunofluorescence signals were visualized under Leica TCS SP5 confocal laser microscope. The antibodies are shown in the **Suppl. Table 1**.

**4.11 Masson’s trichrome staining**

The left mice lungs section (5 µm) was subjected to Masson’s trichrome (ab150686, Abcam, Cambridge, UK) staining according to the manufacturer’s instructions (Chen et al., 2017; Landini et al., 2020).

**4.12 Hydroxyproline colorimetric assay**

Collagen production was investigated by determination of hydroxyproline levels in right lung tissue or in cell homogenates using the Hydroxyproline Assay kit (MAK008, Sigma, Buchs, Switzerland) according to the manufacturer’s instructions.

**4.13 ELISA**

TGF-β1 and IL-1β concentrations in the conditioned medium from human epithelial cells were measured by ELISA kits (human TGF-β1, 88-8350-22, ThermoFisher, Waltham, Massachusetts, USA; human IL-1β, 437004, BioLegend,San Diego, USA) according to the manufacturer’s instructions. In addition, blood was collected from mouse anaesthetized with 5% isoflurane in oxygen and maintained at 1.5% isoflurane during the procedure. Blood was taken with 30G insulin syringe (B. Braun; Melsungen; Germany) from jugular veins. After collection, blood was allowed to coagulate in a tube with gel clot activator (Microvette 500 Z-Gel; SARSTED AG; Nümbrecht; Germany) for 15 minutes followed by centrifugation for 5 minutes at 10’000 x *g* to separate the serum. Serum was kept at -80 degree till TGFβ1 measurement using the ELISA kit as mentioned above.

**4.14 Statistics**

Data are presented as mean ± SD. Data distribution is determined by Kolmogorov-Smirnov test and statistical analysis for normally distributed values was performed with Student’s unpaired t-test or analysis of variance (ANOVA) with Bonferroni post hoc test. For non-normally distributed values, Mann–Whitney test or the Kruskal–Wallis test was used. Differences in mean values were considered significant at a two-tailed p ≤ 0.05.

**References**

Chen, Y., Yu, Q., & Xu, C.-B. (2017). A convenient method for quantifying collagen fibers in atherosclerotic lesions by ImageJ software. *Int J Clin Exp Med, 10*, 14904-14910.

Landini, G., Martinelli, G., & Piccinini, F. (2020). Colour Deconvolution - stain unmixing in histological imaging. *Bioinformatics*. doi:10.1093/bioinformatics/btaa847

Ming, X. F., Rajapakse, A. G., Yepuri, G., Xiong, Y., Carvas, J. M., Ruffieux, J., . . . Yang, Z. (2012). Arginase II Promotes Macrophage Inflammatory Responses Through Mitochondrial Reactive Oxygen Species, Contributing to Insulin Resistance and Atherogenesis. *J Am Heart Assoc, 1*(4), e000992. doi:10.1161/JAHA.112.000992

Shi, O., Morris, S. M., Jr., Zoghbi, H., Porter, C. W., & O'Brien, W. E. (2001). Generation of a mouse model for arginase II deficiency by targeted disruption of the arginase II gene. *Mol. Cell Biol., 21*(3), 811-813.

Xiong, Y., Yu, Y., Montani, J. P., Yang, Z., & Ming, X. F. (2013). Arginase-II induces vascular smooth muscle cell senescence and apoptosis through p66Shc and p53 independently of its l-arginine ureahydrolase activity: implications for atherosclerotic plaque vulnerability. *Journal of the American Heart Association, 2*(4), e000096. doi:10.1161/JAHA.113.000096
