# Supplementary material for: Role of pulmonary epithelial arginase‐II in activation of fibroblasts and lung inflammaging
Source: Aging Cell. 2023 Feb 15;22(4):e13790. doi: 10.1111/acel.13790 (PMC10086530; doi:10.1111/acel.13790)
Supplement: Supplementary file 2 — Table S1. Table S2. [file ACEL-22-e13790-s002.docx]

| **Antibody target** | **Dilution** |
| --- | --- |
| Arg-II (#55003, cell signaling) | WB 1:1000;  IF 1:100 |
| Arg-I (610708, BD Transduction Laboratories) | WB 1:1000;  IF 1:50 |
| SP-C (sc-519029, Santa Cruz) | IF 1:50 |
| CC-10 (sc-365992, Santa Cruz) | IF 1:100 |
| FOXJ1(14-9965-82, Thermo Fisher Scientific) | IF 1:100 |
| TGFβ1 (ab215715, Abcam) | WB 1:1000;  IF 1:200 |
| TGFβ1 (sc-130348, Santa Cruz) | IF 1:100 |
| PDGF-R**α** (AF1062 -R&D Systems) | IF 1:100 |
| IL-1β (ab9722, Abcam) | IF 1:100 |
| a-SMA (ab7817, Abcam) | IF 1:200 |
| β-actin (A5441, Sigma-Aldrich) | WB 1:10,000 |
| GAPDH (10R-G109A,Sigma-Aldrich) | WB 1:10,000 |
| IRDye 800-conjugated affinity purified goat anti-rabbit IgG  (9263221, BioConcept) | WB 1:5,000 |
| Alexa fluor 680-conjugated goat anti-mouse IgG (A-21057, Invitrogen) | WB 1:5,000 |
| Alexa Fluor 488-conjugated goat anti-rabbit IgG (H+L) secondary Ab (A-11008, Thermo Fisher Scientific) | IF 1:400 |
| Alexa Fluor 594-conjugated goat anti-rabbit IgG (H+L) secondary Ab (A-11012, Thermo Fisher Scientific) | IF 1:400 |
| Alexa Fluor 488-conjugated goat anti-mouse IgG (H+L) secondary Ab (A-11001, Thermo Fisher Scientific) | IF 1:400 |
| Alexa Fluor 568-conjugated goat anti-mouse IgG (H+L) secondary Ab (A-11031, Thermo Fisher Scientific) | IF 1:400 |

**Suppl. Table 1. Antibody dilutions used for immunoblotting and immunofluorescence**

**Suppl. Table 2. The RT-PCR primer sequences**

| **The following primer sequences of mouse origin were used:** | | |
| --- | --- | --- |
| **Gene** | **Forward primer sequences (5’-3’)** | **Reverse primer sequences (5’-3’)** |
| *arg-1* | 5′-GGA ATC TGC ATG GGC AAC CTG TGT-3′ | 5′-AGG GTC TAC GTC TCG CAA GCC A-3′ |
| *arg-2* | 5′-CCC CTT TCT CTC GGG GAC AGA A-3′ | 5′-GAA AGG AAA GTG GCT GTC CA-3′ |
| *f4/80* | 5′-TGG CTG CCT CCC TGA CTT TC-3′ | 5′-CAA GAT CCC TGC CCT GCA CT-3′ |
| *il -6* | 5′-GAC AAC CAC GGC CTT CCC TA-3′ | 5′-GCC TCC GAC TTG TGA AGT GGT-3′ |
| *il-1β* | 5′-GCA ACT GTT CCT GAA CTC AAC T-3′ | 5′-TCT TTT GGG GTC CGT CAA CT-3′ |
| *inos* | 5′-GGC AAA CCC AAG GTC TAC GTT-3′ | 5′-TCG CTC AAG TTC AGC TTG GT-3′ |
| *mcp1* | 5′-AGC ACC AGC CAA CTC TCA C-3′ | 5′-TCT GGA CCC ATT CCT TCT TG-3′ |
| *tnf-α* | 5′-GGC AGG TCT ACT TTG GAG TCA TTG C-3′ | 5′-ACA TTC GAG GCT CCA GTG AAT TCG G-3′ |
| *tgf-β1* | 5′-TGG AGC AAC ATG TGG AAC TC-3′ | 5′-CAG CAG CCG GTT ACC AAG-3′ |
| *p16^ink1^* | 5′-GAA CTC TTT CGG TCG TAC-3′ | 5′-GCA GAA GAG CTG CTA CGT-3′ |
| *p21^Cip1^* | 5′-GTC CAA TCC TGG TGA TGT CC-3′ | 5′-GTT TTC GGC CCT GAG ATG T-3′ |
| *rps12* | 5′-GAA GCT GCC AAA GCC TTA GA-3′ | 5′-AAC TGC AAC CAA CCA CCT TC-3′ |
|  | | |
| **The following primer sequences of human origin were used:** | | |
| **Gene** | **Forward primer sequences (5’-3’)** | **Reverse primer sequences (5’-3’)** |
| *arg-ii* | 5′-GGC TGA GGT GGT TAG CAG AG-3′ | 5′-CTG GCT GTC CAT GGA GAT TT-3′ |
| *tgf-β1* | 5′-CCC AGC ATC TGC AAA GCT C-3′ | 5′-GTC AAT GTA CAG CTG CCG CA-3′ |
| *a-sma* | 5′-CCC TTG AGA AGA GTT ACG AGT TG-3′ | 5′-ATG ATG CTG TTG TAG GTG GTT TC-3′ |
| *il-1β* | 5′-TCT TCG ACA CAT GGG ATA ACG A-3′ | 5′-TCC CGG AGC GTG CAG TT-3′ |
| *il -6* | 5′-GGC ACT GGC AGA AAA CAA CC-3′ | 5′-GCA AGT CTC CTC ATT GAA TCC-3′ |
| *tnf-α* | 5′-CCC AGG GAC CTC TCT CTA ATC A-3′ | 5′-GCT ACA GGC TTG TCA CTG GG-3′ |
| *sdha* | 5′-TGG GAA CAA GAG GGC ATC-3′ | 5′-CCA CCA CTG CAT CAA ATT CAT-3′ |
